# Identification of risk factors associated with carriage of resistant *Escherichia coli* in three culturally diverse ethnic groups in Tanzania: a biological and socioeconomic analysis

**DOI:** 10.1016/S2542-5196(18)30225-0

**Published:** 2018-11

**Authors:** Mark A Caudell, Colette Mair, Murugan Subbiah, Louise Matthews, Robert J Quinlan, Marsha B Quinlan, Ruth Zadoks, Julius Keyyu, Douglas R Call

**Affiliations:** a Paul G Allen School for Global Animal Health, Washington State University, Pullman, WA, USA; b Department of Anthropology, Washington State University, Pullman, WA, USA; c Institute of Biodiversity, Animal Health and Comparative Medicine, University of Glasgow, Glasgow, UK; d Tanzania Wildlife Research Institute, Arusha, Tanzania; e Nelson Mandela African Institution of Science and Technology, Arusha, Tanzania

## Abstract

**Backgound:**

Improved antimicrobial stewardship, sanitation, and hygiene are WHO-inspired priorities for restriction of the spread of antimicrobial resistance. Prioritisation among these objectives is essential, particularly in low-income and middle-income countries, but the factors contributing most to antimicrobial resistance are typically unknown and could vary substantially between and within countries. We aimed to identify the biological and socioeconomic risk factors associated with carriage of resistant *Escherichia coli* in three culturally diverse ethnic groups in northern Tanzania.

**Methods:**

We developed a survey containing more than 200 items and administered it in randomly selected households in 13 Chagga, Arusha, or Maasai villages chosen on the basis of ethnic composition and distance to urban centres. Human stool samples were collected from a subset of households, as were liquid milk samples and swabs of milk containers. Samples were processed and plated onto MacConkey agar plates, then presumptive *E coli* isolates were identified on the basis of colony morphology. Susceptibility of isolates was then tested against a panel of nine antimicrobials (ampicillin, ceftazidime, chloramphenicol, ciprofloxacin, kanamycin, streptomycin, sulfamethoxazole, tetracycline, and trimethoprim) via a breakpoint assay. Susceptibility findings were matched with data across a wide range of household characteristics, including education, hygiene practices, wealth, livestock husbandry, and antibiotic use.

**Findings:**

Between March 23, 2012, and July 30, 2015, we interviewed 391 households (118 Arusha, 100 Chagga, and 173 Maasai). Human stool samples were collected at 226 (58%) households across the 13 villages. 181 milk samples and 191 milk-container swabs were collected from 117 households across seven villages. 11 470 putative *E coli* samples were isolated from stool samples. Antimicrobial use in people and livestock was not associated with prevalence of resistance at the household level. Instead, the factors with the greatest predictive value involved exposure to bacteria, and were intimately connected with fundamental cultural differences across study groups. These factors included how different subsistence types (pastoralists *vs* farmers) access water sources and consumption of unboiled milk, reflecting increased exposure to resistant bacteria in milk.

**Interpretation:**

When cultural and ecological conditions favour bacterial transmission, there is a high likelihood that people will harbour antimicrobial-resistant bacteria irrespective of antimicrobial use practices. Public health interventions to limit antimicrobial resistance need to be tailored to local practices that affect bacterial transmission.

**Funding:**

US National Science Foundation; Biotechnology and Biological Sciences Research Council, UK Medical Research Council; and the Allen School.

## Introduction

Antimicrobial use is the ultimate factor driving the emergence of antimicrobial resistance.^1^ This fact is the motivation for policies that are intended to reduce unnecessary antimicrobial use, which is often a focus of WHO-inspired national antimicrobial-resistance action plans around the world.^2^ The tangled interplay of antimicrobial use and microbial transmission between people, animals, and the environment complicates efforts to reduce antimicrobial resistance. These issues are likely to be most acute in low-income and middle-income countries, where laissez-faire access to, and use of, antimicrobials is frequently coupled with a high prevalence of infectious disease.^3^, ^4^ Although many studies have examined risk factors for antimicrobial resistance,^2^ they have typically focused on ultimate selective factors (eg, antimicrobial use, health-care-seeking behaviour) and have often been done in clinical contexts.^1^ Comprehensive examinations of a range of ultimate and proximate drivers of antimicrobial resistance at the community level have been done in only a few studies.^5^, ^6^, ^7^, ^8^, ^9^ To our knowledge, no studies have been published in which a cross-cultural sampling design was used to examine how factors can vary across communities—especially when these communities are likely to interact to some degree. This is a crucial information gap, particularly in low-income and middle-income countries, where communities collectively have an extraordinary diversity of socioeconomic systems that blend western and traditional medical belief systems and that operate within varying regulatory and health-care environments.

**Research in context**

**Evidence before this study**

We searched PubMed and Google Scholar with the terms “antimicrobial resistance” OR “antibiotic resistance” AND “community” AND “risk factors” OR “correlates” for reports of studies done in low-income or lower-middle-income countries that combined household-level surveys with collection of biological samples from people published in English up to May 5, 2018. Our search identified five relevant studies. Two showed evidence that antimicrobial resistance was associated with antibiotic use. Other risk factors identified in the studies included hygiene practices such as handwashing and the availability of potable water sources, and socioeconomic factors including household wealth, household density, and maternal literacy. Combination of the findings from these five studies to compare effect sizes for the different risk factors is complicated, however, because of differences in both questionnaires (eg, some questionnaires seemed largely limited to questions about antibiotic use) and laboratory methods used to measure antimicrobial susceptibility. We did not find any studies in which a cross-cultural approach was explicitly used to examine risk factors for antimicrobial resistance.

**Added value of this study**

In this study, we combined extensive collection of bacterial isolates (more than 11 000) with collection of comprehensive data for household-level health and hygiene practices and socioeconomic characteristics. By using the same data-collection and data-analysis protocols across three culturally diverse ethnic groups, we were able to assess the relative contribution of livelihood dimensions (eg, animal husbandry practices, engagement with modern human and animal health sectors, hygiene practices) to community patterns of antibiotic resistance. In line with two previous studies, we found that antibiotic resistance in *Escherichia coli* was not significantly associated with antibiotic use. Instead, we showed that the prevalence of antibiotic-resistant *E coli* was associated with cultural–ecological factors that affect bacterial transmission, including how water is accessed and how milk is handled. Although studies have shown the presence of resistant pathogens in milk from households in low-income countries, to our knowledge, our study is the first to show that consumption of milk is associated with a dose-dependent increase in the risk of people carrying antimicrobial-resistant *E coli*. The mechanism appears to be mostly associated with consumption of antibiotic-resistant bacteria in milk.

**Implications of all the available evidence**

Local, national, and global policies and plans that emphasise the importance of antibiotic stewardship rather than bacterial transmission might have little near-term effects on the prevalence of antibiotic-resistant bacteria in low-income countries. Because of funding constraints and difficulties in drug regulation in these countries, interventions that promote behavioural changes that limit transmission, including practices related to hygiene and sanitation, should be prioritised. Many of these practices will be culturally framed (eg, through ethnomedical belief systems), ensuring that attention should be given to the social context in which antimicrobial-resistant organisms are favoured and transmitted.

To better understand the complexity and interactions of factors driving antimicrobial resistance across pastoralist and impoverished communities in low-income and middle-income countries, we aimed to identify risk factors associated with the carriage of resistant *Escherichia coli* in three ethnic groups in northern Tanzania.

## Methods

### Cultural background

We did a mixed-methods study that combined extensive ethnographic observations with collection of biological samples to identify factors associated with carriage of antimicrobial-resistant *E coli* in Chagga highland farmers, Maasai pastoralists, and Arusha agropastoralists in Tanzania (appendix). These communities vary considerably across social dimensions that have been routinely proposed as potential risk factors for antimicrobial resistance (appendix),^10^ including education and income levels, antimicrobial use and access, animal husbandry practices, proximity to urban environments, and hygiene and sanitation practices. However, the Chagga, Maasai, and Arusha also live in close proximity to each other, such that households buy and sell livestock and crops at the same markets, visit the same hospitals and pharmacies, and deal with the same regulations and policies that guide human and animal health care in Tanzania. Differences between communities could drive differences in the prevalence of antimicrobial resistance, but it is possible that interactions between communities will diminish the effects of cultural and ecological differences.

Chagga farmers live on the slopes of Mount Kilimanjaro in close proximity to the major urban centre of Moshi, which has a population of around 200 000 people. Most Chagga households rely on mixed agriculture, with small zero-grazing (ie, animals are confined to pens and brought fodder and water) herds of small stock (ie, sheep and goats; mean number of animals=4) and cattle (mean number of animals=1·5).^10^ Other Chagga households derive most of their income from waged and salaried jobs in Moshi and surrounding areas,^11^ and rely on professional veterinary care. Almost every Chagga household (96%) reported adherence to antibiotic-withdrawal periods before consumption of meat and milk.^10^ Approximately 92% of Chagga household heads have received some formal education.^10^

Maasai pastoralists live throughout Tanzania and Kenya.^12^, ^13^, ^14^ Most Maasai supplement their main herding livelihoods by growing crops (mostly maize and beans).^12^ In the six Maasai villages surveyed (appendix), households had a mean of 84 cattle and 142 small stock.^10^ More than 70% of Maasai household heads had no formal education.^10^ Most Maasai households (74%) regularly diagnose disease in their animals and administer veterinary medicines without veterinary supervision, and most people (93%) were unlikely to adhere to withdrawal periods after giving veterinary antibiotics to their animals.^10^

Arusha agropastoralists inhabit the periurban areas surrounding Arusha, a major municipality in northern Tanzania with a population of around 420 000. Arusha tend to combine smallholder cultivation of beans, bananas, and coffee with small-scale livestock keeping.^15^ In the five Arusha villages surveyed, small-stock herds contained a mean of six animals and cattle herds contained a mean of two animals.^10^ 30% of household heads had no formal education.^10^ Shared history with the Maasai could account for why 40% of Arusha households administer antibiotics to their animals without veterinary supervision, even though their subsistence patterns, access to modern veterinary services, and distance to urban areas are more similar to those of the Chagga.^10^, ^15^ Only 28% of Arusha reported adherence to veterinary antibiotic-withdrawal guidelines.^10^

### Procedures

A mixed-methods, qualitative and quantitative approach was used to develop the survey instrument, which included 226 items (appendix). To measure antibiotic use, we included self-reported indicators of lay use, health-care visits, the number of antibiotics or packages in the household, and ownership of syringes and needles. Previous work showed that a combination of self-reported and direct observation offered a robust scale of antimicrobial use (appendix).^10^ The study was reviewed and approved by the Washington State University (IRB12355) and Tanzania National Institute for Medical Research institutional review boards, and then the Tanzania Commission for Science and Technology subsequently issued a research permit (permit 2012-151).

We purposefully selected 13 villages on the basis of ethnic composition and distance to urban centres (which could affect prevalence of antimicrobial resistance;^16^, ^17^, ^18^ appendix). Although villages were purposefully chosen, households were randomly selected from census lists provided by local offices. Surveys were administered to the household head by research assistants who were fluent in English, Swahili, and Maa or Chagga, and who received 1 month of training. We collected human stool samples when available at the households. We also started to collect liquid milk and swabs of milk containers from Maasai and Arusha households after initial results suggested a possible association between antimicrobial resistance and milk consumption.

Samples were placed into a portable 12v refrigerator set at 5°C and were transported to the Nelson Mandela African Institution of Science and Technology (Arusha, Tanzania), within 48 h of collection. Faecal samples were diluted in sterile water (1:10 weight per volume). Swab suspensions were made by squeezing swabs inside a whirl-pack with 5 mL of sterile water. Samples and milk were then plated onto MacConkey agar plates using sterile glass beads and were incubated overnight at 37°C. Up to 48 presumptive isolates per sample were selected on the basis of colony morphology that was consistent with *E coli*. This number of isolates provides a greater than 50% probability of detecting at least one isolate with resistance to a given antibiotic when that population of resistant bacteria makes up only 2% of isolates in the sample. Isolates were inoculated individually into the wells of 96-well assay plates containing 150 μL per well of Luria broth. Approximately 40 μL of these cultures were transferred to new 96-well plates, air-dried, and shipped to Washington State University (Pullman, WA, USA) for antimicrobial-susceptibility testing. Serial plating onto MacConkey agar plates was used to estimate the bacterial load (log_10_) of lactose-fermenting bacteria per mL of milk.

Susceptibility to nine antimicrobials was measured with a breakpoint assay. Briefly, MacConkey agar was prepared with fixed concentrations of the antibiotics ampicillin (32 μg/mL), ceftazidime (8 μg/mL), chloramphenicol (32 μg/mL), ciprofloxacin (4 μg/mL), kanamycin (64 μg/mL), streptomycin (16 μg/mL), sulfamethoxazole (512 μg/mL), tetracycline (16 μg/mL), or trimethoprim (8 μg/mL). Concentrations were guided by the Clinical and Laboratory Standards Institute minimum inhibitory concentrations for *Enterobacteriaceae*.^19^ Shipped bacteria were recovered from a desiccated state by adding 150 μL Luria broth, followed by stationary incubation overnight (37°C). We used a 96-pin replicator to transfer culture (approximately 2 μL) onto agar media plates, which were subsequently incubated overnight. A positive (resistant) and negative (sensitive) control strain of *E coli* was added to every plate. Cultures that grew full colonies were considered to be antibiotic resistant. Cultures for which there was little to no growth were scored as antibiotic sensitive.

To investigate whether single boiling events can affect the biological activity of oxytetracycline residues in milk, different concentrations of oxytetracycline were added to aliquots of milk that were subsequently heated to boiling temperature for 2–3 min. After cooling to room temperature, oxytetracycline-resistant or oxytetracycline-susceptible *E coli* cultures (isogenic strains) were added to each aliquot (6 log_10_ colony-forming units per mL per strain). These cultures were then incubated at 37°C for 24 h, after which the numbers of colony-forming units of resistant and susceptible *E coli* were enumerated on MacConkey agar plates to establish which strain dominated the culture (appendix).

### Statistical analysis

To identify the risk factors associated with antimicrobial resistance, we compiled a set of 56 variables from our household surveys (appendix) that detailed health-care practices and human and livestock antibiotic use. These variables have been previously proposed or identified as risk factors for antimicrobial resistance.^2^, ^3^, ^4^, ^5^, ^6^, ^7^, ^8^, ^9^, ^10^ Random Lasso regressions^20^ (appendix) were used to identify variables associated with resistance to one of the five most prevalent antimicrobial-resistance phenotypes (ampicillin, streptomycin, sulfamethoxazole, tetracycline, and trimethoprim) or with resistance to three or more antibiotics (multidrug resistance; appendix). The Lasso regressions functioned as a variable-selection process, which reduced the original 56 variables to a subset of variables (appendix) that were consistently related to antimicrobial resistance. Variables that were excluded by the Lasso regressions were considered not to be significant. Variable subsets were then entered into mixed-effects logistic regression models (clustered at the household). We calculated odds ratios (ORs) for the best-fit models (assessed with the Akaike information criterion).

### Role of the funding source

The study funders had no role in study design; data collection, analysis, or interpretation; or writing of the report. The corresponding author had full access to all study data and had final responsibility for the decision to submit for publication.

## Results

Between March 23, 2012, and July 30, 2015, we interviewed 391 households (118 Arusha, 100 Chagga, and 173 Maasai). Human stool samples were collected at 226 (58%) households across the 13 villages (three Arusha, four Chagga, and six Maasai). 181 milk samples and 191 milk-container swabs were collected from 117 households across seven villages (three Arusha and four Maasai).

**Figure 1:**
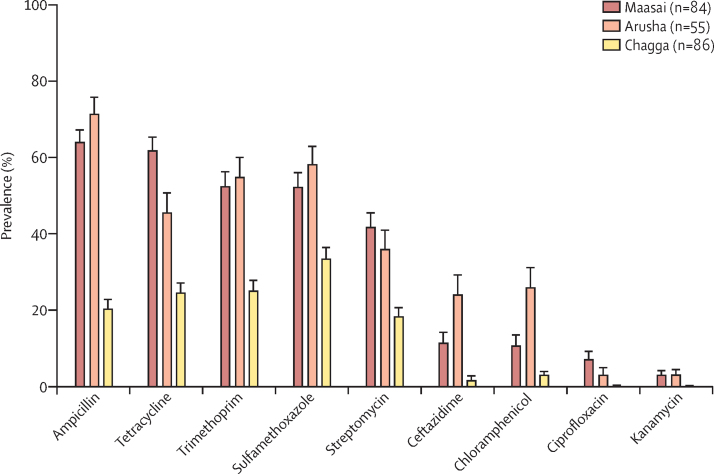
Mean household prevalence of antimicrobial-resistant *Escherichia coli* isolated from stool samples collected from Maasai, Arusha, and Chagga households Data are the number of households. Error bars represent SE. Between-group comparisons (including statistical comparisons) for the prevalence of resistant bacteria are in the appendix.

11 470 putative *E coli* samples were isolated from stool samples (appendix). The mean prevalence of resistance to the nine antimicrobials tested for was highest for Maasai and Arusha households, and lowest for Chagga households (figure 1). The prevalence of resistance to ampicillin, tetracycline, trimethoprim, sulfamethoxazole, and streptomycin was consistently higher than that to other tested antibiotics across all groups (figure 1). The diversity of resistance phenotypes was similar across groups (appendix).

For the Chagga, no factor was significantly associated with the odds that bacteria would be resistant to more than one antimicrobial (appendix). Households reporting that they “interrupted their business” (eg, stopped going to work) to care for sick livestock were less likely to carry tetracycline-resistant *E coli* than those reporting no interruption (OR 0·34 [95% CI 0·14–0·66]). An increased number of water sources used for livestock was associated with an increased likelihood of trimethoprim resistance (1·89 [1·10–2·78]). Arusha households that shared water sources with livestock and wildlife were far more likely to carry multidrug-resistant *E coli* (7·54 [2·41–23·45]), and *E coli* resistant to sulfamethoxazole (9·23 [3·19–26·75]), streptomycin (11·70 [2·74–50·01]), and tetracycline (6·23 [2·05–19·02]) than were households that shared water sources with livestock only (appendix).

**Figure 2:**
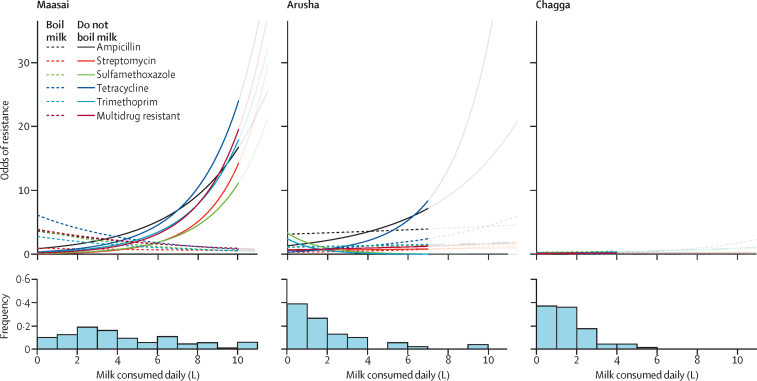
Odds of carriage of *Escherichia coli* resistant to ampicillin, tetracycline, trimethoprim, sulfamethoxazole, and streptomycin, and of multidrug-resistant *E coli*, relative to milk consumption for households that boil and do not boil milk Lines shown as transparent are for values exceeding two SDs above the mean milk consumption (x-axis) for the ethnic group. Odds of resistance refers to the odds that an isolate from a household exhibited resistance to that particular antibiotic. On the y-axis of the histograms, “Frequency” refers to the proportion of households. Other antibiotics were not included in the models because of an overall low prevalence across ethnic groups (figure 1).

For Maasai households, consumption of unboiled (raw) milk was associated with increased odds of carriage of *E coli* resistant to each antibiotic and of multidrug-resistant *E coli* (figure 2; appendix). When we compared household milk consumptions 25% below (ie, 1·1 L) the mean with those 25% above (ie, 6·0 L) the mean, the odds of detecting resistance increased from 1·48 (95% CI 1·10–2·01) to 8·67 (6·42–11·70) for ampicillin, from 1·62 (1·19–2·28) to 14·28 (10·44–19·54) for streptomycin, from 1·75 (1·26–2·42) to 21·33 (15·46–29·43) for sulfamethoxazole, from 1·56 (1·17–2·10) to 11·70 (8·74–15·70) for tetracycline, from 1·52 (1·12–2·07) to 9·94 (7·29–13·57) for trimethoprim, and from 1·54 (1·16–2·02) to 10·38 (7·89–13·66) for multidrug resistance. Assuming that increased antibiotic use translates into a higher prevalence of antimicrobial-resistant bacteria,^1^ then the strong correlation between antimicrobial resistance and consumption of raw milk in the Maasai could be explained by increasing milk-borne illness and subsequent antibiotic use. Indeed, Maasai families frequent health clinics 11% more often than Arusha families, and 27% more often than Chagga families.^10^ Nevertheless, our Lasso variable-selection process showed no consistent relationship between antimicrobial use and the odds of finding antimicrobial-resistant *E coli* in Maasai stool samples (appendix).

**Figure 3:**
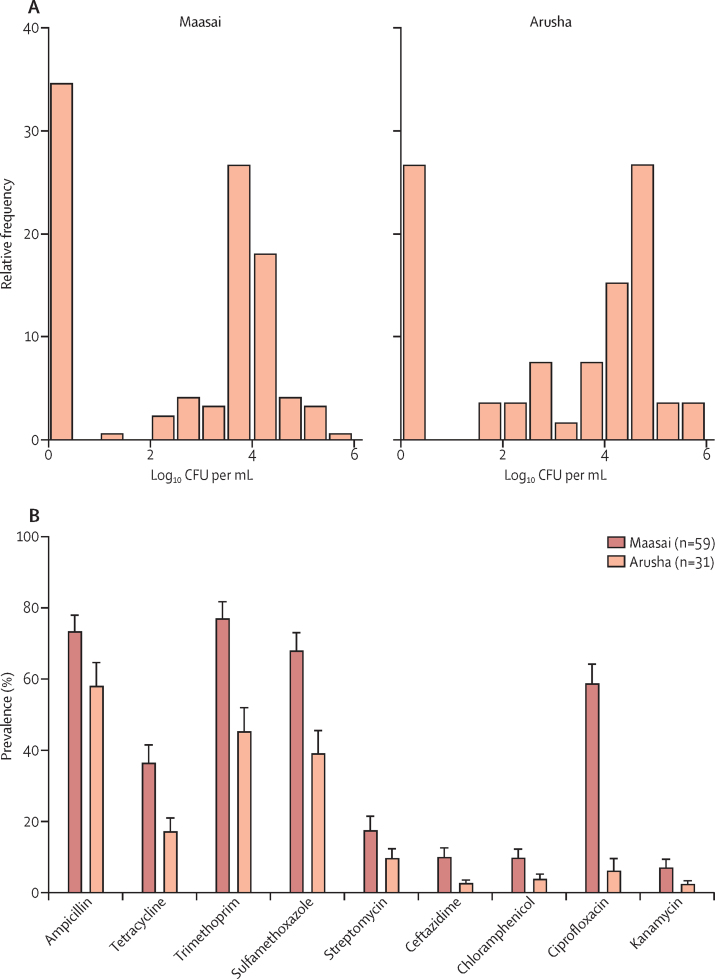
Gram-negative lactose-fermenting bacteria load (A), and mean prevalence of antimicrobial-resistant, Gram-negative lactose-fermenting bacteria (B), in milk samples from Maasai and Arusha households Milk was not sampled in Chagga households because initial models did not identify milk consumption as a risk factor. Figure 3A is based on 167 milk samples from 80 Maasai and 30 Arusha households. Data for figure 3B are the number of households. Error bars in 3B represent SE. Milk from Maasai households harboured significantly (according to single-factor multivariate ANOVA) more ciprofloxacin-resistant (p<0·0001), sulfamethoxazole-resistant (p=0·003), tetracycline-resistant (p=0·03), and trimethoprim-resistant (p=0·001) bacteria than that from Arusha households. CFU=colony-forming units.

**Figure 4:**
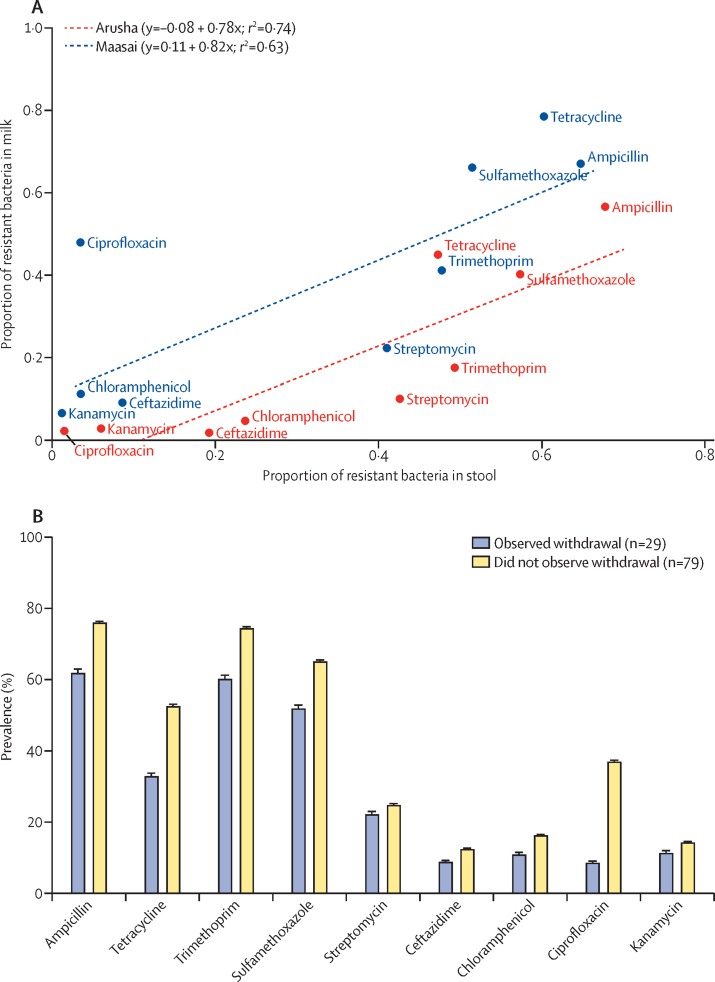
Proportion of resistant *Escherichia coli* in stool samples relative to proportion of resistant lactose-fermenting Gram-negative bacteria in stored milk samples (A), and mean prevalence of resistant lactose-fermenting Gram-negative bacteria in milk samples from Maasai and Arusha households that did and did not adhere to antibiotic-withdrawal guidelines before milk consumption (B) Milk was not sampled in Chagga households because initial models did not identify milk consumption as a risk factor. Data in figure 4A are paired at the household level (31 Maasai households and 19 Arusha households), although samples were collected on different dates. Figure 4B represents data for 8106 isolates from 167 samples. The figure shows the number of households. Multivariate ANOVA showed that milk sampled from households that adhered to withdrawal guidelines had a significantly lower prevalence of antimicrobial-resistant bacteria for all antimicrobials tested except for ampicillin compared with samples from households that did not adhere to withdrawal guidelines (appendix).

The relationship between milk and antimicrobial resistance could also be because of consumption of antibiotic-resistant bacteria in contaminated milk. 73 (63%) of 115 milk samples from 59 Maasai households and 37 (71%) of 52 samples from 31 Arusha households had counts of lactose-fermenting bacteria (including *E coli*) between 10^2^ and 10^6^ per mL (figure 3A). Although *E coli* is lactose-fermenting, the proportion of *E coli* in these samples was low (21·7% in milk samples, and 9·5% in milk-container swabs; appendix). Nevertheless, when we compared *E coli* isolates that were confirmed by DNA sequencing with other lactose-fermenting bacteria (*Enterobacter* spp and *Klebsiella* spp; appendix), there was a high correlation (*r*^2^=0·92) in terms of the prevalence of resistance to all nine antibiotics. There was also a strong correlation between the proportion of antimicrobial-resistant lactose-fermenting bacteria detected in milk samples (figure 3B) and stool samples for household-matched samples (figure 4A; appendix). Resistance to several antibiotics was more prevalent in lactose-fermenting bacteria isolated from Maasai milk samples than in those isolated from Arusha milk samples (figure 3B).

The higher proportion of antimicrobial-resistant lactose-fermenting bacteria in Maasai milk could have arisen from the presence of antibiotic residues in the milk, because 93% of Maasai households do not adhere to withdrawal times for veterinary antibiotics, compared with 28% of Arusha households and 4% of Chagga households.^10^ We did not measure concentrations of antibiotic residues, but the prevalence of antimicrobial-resistant lactose-fermenting bacteria in milk was 5–10% lower for households that consistently adhered to withdrawal times compared with those that were willing to consume contaminated milk (figure 4B) and for households that used more antibiotics compared with those who used less (appendix), and 3–5% lower (except for ceftazidime) for households that did not consume milk from sick cows that might have been treated with antibiotics compared with those that did (appendix). Despite this association, adherence to antibiotic-withdrawal periods before milk consumption did not significantly affect prevalence according to our Lasso regression procedure (appendix). If there were a drug-residue effect, it would most likely be associated with oxytetracycline, because approximately 80% of Maasai households report recent use of this antibiotic.^10^ If the bioavailability of oxytetracycline was reduced by boiling milk, this would strengthen the possible relationship with consumption of raw milk. According to our results, boiling milk was unlikely to reduce the bioavailability of oxytetracycline residues (appendix). Nevertheless, for households that adhered to withdrawal periods, approximately 30% of milk samples were positive for tetracycline-resistant lactose-fermenting bacteria (figure 4B). For households that did not adhere to withdrawal periods, approximately 53% of the milk samples were tetracycline-resistant lactose-fermenting bacteria (figure 4B).

## Discussion

In this mixed-methods study, we showed that, although the relative patterns of resistance to nine antibiotics in *E coli* carried by Chagga, Arusha, and Maasaia peoples in northern Tanzania are generally similar, there are substantial differences in prevalence across the three groups, which are linked to group-specific risk factors. Contrary to conventional expectations, differences in prevalence were not associated with antibiotic use in animals or people but were instead associated with culturally informed practices that affect bacterial transmission, including livestock management and food preferences. Our results emphasise that efforts to address antimicrobial resistance globally will require approaches that address the drivers of resistance within the cultural context of communities.

The prevalence of *E coli* resistant to ampicillin, tetracycline, trimethoprim, sulfamethoxazole, or streptomycin, all of which are used in human medicine, was high in Maasai and Arusha households. Although the breakpoint assay that we used is different from the clinical microbiology assays used in many other studies of antimicrobial resistance studies, our findings are similar to those of other studies in low-income settings. For example, community sampling in Peru found similar proportions of *E coli* resistant to ampicillin (62·6%), cotrimoxazole (48·6%), tetracycline (43·0%), and chloramphenicol (15·8%).^6^, ^21^, ^22^ These community-level estimates are based on randomly selected samples and we should expect these estimates to be lower than estimates from non-random, clinical datasets.^21^ For example, *Shigella* spp, *Salmonella* spp, and *E coli* isolated from children presenting with acute diarrhoea in western Kenya were highly resistant to ampicillin (81·3%), tetracycline (75·0%), and cotrimoxazole (92·8%).^5^

Neither human nor veterinary antibiotic use was a significant predictor of the carriage of antimicrobial-resistant *E coli* in any ethnic group. This finding does not mean that antibiotic use has no effect, but rather that practices affecting transmission were more important drivers of the prevalence of resistance. Water was a risk factor for both Chagga and Arusha. For Chagga households, each additional water source for livestock increased the chances of people having trimethoprim-resistant *E coli*. Bacteria with similar resistance patterns to those in this study were found in waters in the same area in another study.^22^ Chagga usually access drinking water from sources without animal contact, and thus the association with livestock water access could reflect contact transmission at the household level. The odds of carrying resistant *E coli* was increased when Arusha households shared water sources with larger livestock herds and wildlife, which probably reflects both contact and water-mediated transmission to people.

For Maasai households, the association between antimicrobial-resistant *E coli* and volume of milk consumption was consistent across the analysed resistance phenotypes. Importantly, although there was an association between carriage of these bacteria and antimicrobial-resistant bacteria in Arusha milk, milk consumption was not an important factor for Arusha households. This finding probably relates to the fact that Maasai households consume substantially more milk daily (mean 3·92 L) than do Arusha (1·67 L) or Chagga households (1·20 L). Consumption of raw milk is a risk factor for disease transmission,^23^ and thus it is also likely to be a source for transmission of antimicrobial-resistant bacteria (whether pathogenic or not). Pathogen transmission could lead to greater demand for antibiotics by people, although our self-reported data did not show this association.

The mechanism underlying the association between milk and antimicrobial resistance appears to be related to basic transmission. This association has important implications for low-income countries, where potable water remains a pressing challenge,^24^ consumption of dairy products is increasing, and most milk is produced and distributed through informal sources that operate outside national quality-control standards and regulations.^21^ More than 60% of milk samples that we tested had up to 6 log_10_ mL bacteria, and the distribution of resistance phenotypes was similar to that in stool samples for both milk and swabs of milk-storage containers (Figure 3, Figure 3; appendix). Antibiotic withdrawal could contribute to this pattern if residues are present in milk. Our ability to detect a statistical association was limited in Maasai households because only 7% adhered to the recommended antibiotic-withdrawal period. There was more variation in Arusha households, with 72% adhering to recommended withdrawal periods. Nevertheless, for oxytetracycline, the most common veterinary antibiotic in use, the pattern was the opposite of what would be predicted, with approximately 30% of lactose-fermenting bacteria isolates resistant to tetracycline when antibiotic withdrawal was not adhered to and roughly 53% of isolates resistant when households adhered to withdrawal guidelines. It is possible that drug residues could be consumed and select for resistant bacteria in people, but concentrations in milk rarely exceed 1 part per million for oxytetracycline,^25^ and dilution, adsorption, and dissipation are likely to further limit the potential effect on the gut flora of the person ingesting the milk. Finally, although there is some evidence for possible withdrawal effects (figure 4B), this effect is quite small compared with the effects of milk consumption. For example, consuming 1 L of milk containing 4 log_10_ bacteria per mL translates into an inoculum of 10 million bacteria, many of which harbour resistance to antimicrobials.

As a result, the most probable mechanism underlying the raw milk association is dose-dependent oral transmission. Why antimicrobial-resistant bacteria are found in milk in remotely located pastoral communities is worth examining. Veterinary antibiotics could have a role in this process, but the sporadic nature of their use relative to a potential equilibrium of resistance in the population could confound attempts to establish a statistical relationship. Nevertheless, we surmise that sporadic antibiotic use, when coupled with non-adherence to antibiotic-withdrawal periods and poor hygiene and sanitation, leads to periodic amplification of resistant bacteria in milk and milk-storage vessels. Because resistance traits rarely induce a substantial fitness cost,^26^ resistant and susceptible bacteria are generally equally competitive, and therefore only sporadic amplification (or introductions from outside sources) might be needed to maintain a high prevalence of resistant bacteria in a population.

Our study has several limitations. Self-reported data can be inaccurate because of recall and social desirability biases, which, if biased by ethnic group, could lead to spurious correlations.^27^ Furthermore, like any cross-sectional study, correlations cannot prove causation because unmeasured variables could be covarying with the ones that we used in the analysis. For example, when we pooled data across all households, ethnic group was the strongest predictor of differences in the prevalence of resistance (appendix). This finding was consistent with the shared cultural history within groups representing the aggregate driver of differences between groups. Important aspects of this shared history probably include patterns of antibiotic use, disease, food traditions, and animal–human connectivity that combine to establish different baseline levels of resistance. In view of the contribution of this shared history, we used separate models for each ethnic group. Additionally, although risk factors for antimicrobial resistance in the Arusha and Maasai were associated with multiple antibiotics, the few risk factors in the Chagga were only associated with one antibiotic, which increases the chance for spurious associations. The microbiology methods used in this study were the most practical given logistic realities,^28^ but we acknowledge that identification of *E coli* by colony morphology only could lead to misidentification. In an associated whole-genome-sequencing project based on a subset of bacteria from this study, 9% of isolates from human stool samples presumed to be *E coli* were actually *Enterobacter* or *Klebsiella* species (appendix). Importantly, we found no evidence that there was bias in the distribution of these misidentified strains, and the correlation between resistance phenotypes for *E coli* and non-*E coli* isolates was very high (*r*^2^ =0·92).

In conclusion, the primary predictors of the prevalence of antimicrobial resistance in three Tanzanian groups were related to transmission rather than direct selection for resistant bacteria. Our findings emphasise the need to devote more resources to improvement of hygiene and sanitation, which has been acknowledged as a “neglected aspect in the fight against antimicrobial resistance”.^29^ Furthermore, antimicrobial resistance should be treated as much as a cultural challenge as as a medical one because interventions in low-income and middle-income communities need to target behaviours that affect transmission, some of which will be culture specific. Without careful consideration of the local proximate drivers of transmission, such as milk handling and livestock practices, WHO-inspired national antimicrobial-resistance plans will probably have little effect on resistance in low-income and middle-income countries, where more than 80% of the world's population reside.
