# Supplementary material for: Identification of risk factors associated with carriage of resistant Escherichia coli in three culturally diverse ethnic groups in Tanzania: a biological and socioeconomic analysis
Source: Lancet Planet Health. 2018 Nov;2(11):e489–97. doi: 10.1016/S2542-5196(18)30225-0 (PMC6215761; doi:10.1016/S2542-5196(18)30225-0)
Supplement: Supplementary appendix [file mmc1.pdf]

THE LANCET
Planetary Health

Supplementary appendix

This appendix formed part of the original submission and has been peer reviewed. We post it as supplied by the authors.

Supplement to: Caudell M A, Mair C, Subbiah M, et al. Identification of risk factors associated with carriage of resistant *Escherichia coli* in three culturally diverse ethnic groups in Tanzania: a biological and socioeconomic analysis. *Lancet Planet Health* 2018; **2:** e489–97.

Identification of risk factors associated with carriage of resistant *Escherichia coli* in three culturally diverse ethnic groups in Tanzania: a biological and socioeconomic analysis

Authors: Mark A Caudell, Colette Mair, Murugan Subbiah, Louise Matthews, Robert J Quinlan, Marsha B Quinlan, Ruth Zadoks, Julius Keyyu, and Douglas R. Call

Appendix

1. Materials and Methods

a. Study Design

We rely heavily from Caudell et. al [1] in our discussion of Materials and Methods where a more thorough treatment of study development can be found. A team of veterinarians, livestock extension officers, microbiologists, ecologists, epidemiologists, social scientists, and local community members negotiated and coordinated the research design, planning, logistics, project implementation, data management, and analysis. Key informants and survey participants were compensated 10,000 Tanzanian Shillings (about $6·50 USD) for participation (up to 120 min). Research assistants fluent in English, Swahili and Maa or Chagga were trained and employed (Maasai assistants in Maasai and Arusha villages and Chagga assistants in Chagga villages) for data collection and to facilitate participation. Surveys were conducted in *Maa* among Maasai and in *Kiswahili* among Chagga and Arusha. The study was reviewed by Washington State University's and Tanzania National Institute for Medical Research's Review Boards, and research permits were issued by the Tanzania Commission for Science and Technology, Tanzania Wildlife Research Institute, and by regional, district, and ward offices within Arusha, Manyara, and Kilimanjaro regions.

b. Survey Development

We used a mixed-methods strategy that combined qualitative and quantitative data collection beginning with 20 formal, qualitative, key informant and focus group interviews among Maasai and Chagga livestock owners across a range of communities in 2012. We interviewed livestock extension officers and veterinarians in different communities to determine common practices in different areas and the course of professional veterinary training in Tanzania. We used data from initial interviews to refine our survey instruments. We observed livestock management and veterinary care in multiple Maasai and Chagga households from 2012 to 2015 including use of veterinary antimicrobials, chemical dips, and traditional treatments; necropsy of recently deceased animals, slaughter and butchering, milking and milk handling, breeding, birthing, branding, grazing and fodder provision, and castration. Informal interviews were conducted during the course of direct observation, including conversations that recounted people's health experiences, those of their livestock, and additional detail on the circumstances surrounding illness events. Iterative qualitative interviewing helped to add or modify existing survey instruments as different ethnic groups were studied and as lab results of fecal and milk samples suggested new items for inclusion. We visited multiple animal drug shops in Monduli, Simanjiro, Arusha City and Moshi districts, and interviewed attendants about veterinary antibiotic sales and recommended usage. We held three community meetings after quantitative data collection was complete in those communities. Meetings served as focus groups and opportunities to report our preliminary results. These meetings also allowed us to discuss public health solutions relevant to the study communities. Unless otherwise indicated, our ethnographic description of livestock management and veterinary practices draws on these materials. Formal survey collection began in 2013 and ended in 2015.

1.1. Sampling

Focal villages with sampling season and year are provided in Table S1. Our sample is biased towards data from the dry seasons because of transportation difficulties in the wet season combined with the travel constraints associated with timely processing of milk and fecal specimens.

Table S1. Number of households surveyed by year, village/ward, season, and ethnicity.

Ethnicity	Village/Ward	Season	Year(s)
Maasai (N=)	Monduli	Wet&Dry	2013/15
	Loibor Siret	Dry	2013/15
	Terat	Wet	2013
	Nadonjukin	Wet&Dry	2013/14/15
	Loliondo	Dry	2013
	Komolo	Dry	2015
Arusha (N=58)	Aremeru ward	Wet&Dry	2013/15
	Loroi	Dry	2015
	Meliot	Dry	2015
Chagga (N=85)	Masaera kati	Wet&Dry	2014
	Masaera juu	Dry	2014
	Mamsera chini	Dry	2014
	Mamsera juu	Dry	2014

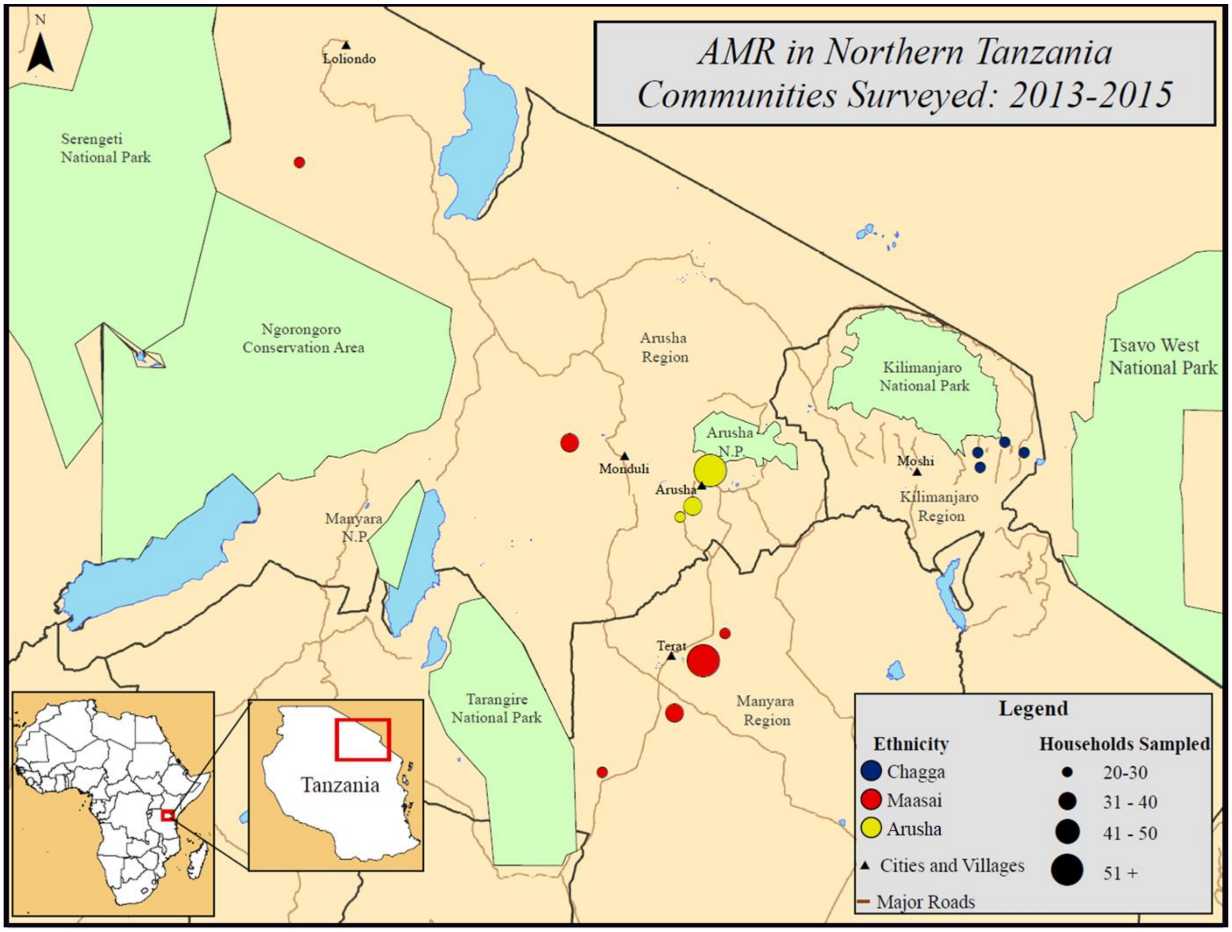

Figure S1. Map of study location with number of households sampled per village.

2. Lab Methods

2.1. Collection and storage of fecal and milk samples

Fecal samples (N=1 per household) were collected from the floor or from the pit toilets and placed into sterile Whirl-Paks. In households with pit toilets (almost every house that had a toilet had drop toilets) a cotton tipped applicator was lowered into the pit. Milk and swab samples were only collected in the final year of survey given preliminary results indicating high levels of resistance in households that consumed large quantities of unboiled milk. A total of 272 milk (n=181) and swab (n = 91) samples were collected across Maasai (n=80) and Arusha households (n=36). Milk samples from cattle, sheep, and goats were collected by pouring milk from collection and storage containers into sterile 50-mL polypropylene tubes. Swab samples were collected by scrubbing the inside of milk containers using moist (~5 mL of water) cotton pads. Swabs were then placed into sterile 50 mL polypropylene tubes. Type of storage containers (calabash, metal, plastic), storage time (if known), and milk type (fresh/sour) were documented. Milk and swab samples were placed into a portable refrigerator that was kept below 5°C and transported to the Nelson Mandela African Institution of Science and Technology (NM-AIST) in Arusha, Tanzania. Upon reception at NM-AIST, the fecal samples were diluted in approximately 9 parts of sterile water; glycerol was added (15% volume/volume final concentration) to a 1-mL aliquot of fecal slurries and were stored at -80˚C.

2.2. Isolation and shipping of putative E. coli

The fresh fecal slurry was serially diluted into sterile water and multiple dilutions were plated onto MacConkey agar (Becton, Dickinson and Company, Sparks, MD) plates using sterile glass-beads for the isolation of presumptive *E. coli* isolates (based on colony morphology). We normally picked 48 isolates per sample, although there was some variation due to limited recovery or when houses were sampled more than once (Fig. S2A). This methodology generated a comparable degree of phenotypic diversity between groups (Fig. S2B, see antibiotic testing details below). Isolates were picked by by using sterile tooth-picks and inoculating these individually into the wells of 96-well assay plates containing 150 µL/well of LB (Luria-Bertani, Becton, Dickinson and Company, Sparks, MD) broth. After inoculation, the plates were covered with lids and wrapped in cling-wrap papers and incubated overnight at 37˚C. After incubation on MacConkey plates. glycerol was added to the original plates and stored at -80˚C. These isolates were shipped to the laboratory at Washington State University in Pullman, WA using the following protocol: a duplicate 96-well culture plate containing 40 µL of LB broth was prepared using 96-pin replicator. These plates were incubated for 10-12 h at 37˚C; the lids were removed and the plates with culture were air-dried in the incubator for up to 24 h. The plates containing desiccated cultures were covered with lid, stacked, put in the cardboard boxes and left at the room temperature for 1-2 weeks. The plates were checked for condensation during this storage period and, if absent, were then shipped to Washington State University (WSU, Pullman, WA, U.S.A.). If condensation was noted, plates were disposed and prepared again.

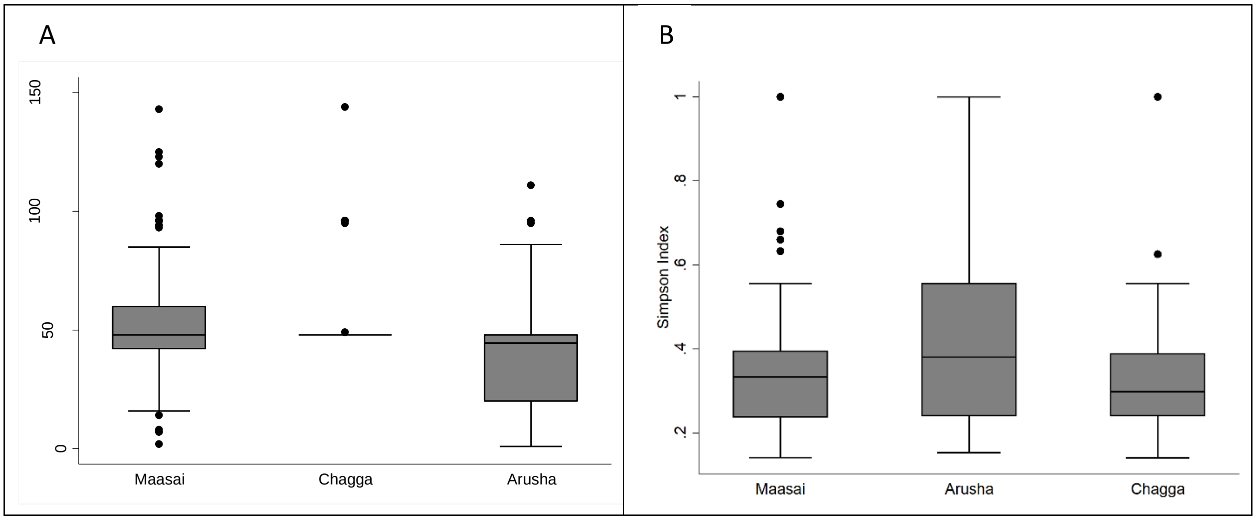

Figure S2. A) Box plots of isolates per household across ethnic groups. B) Box plots of Simpson's Index across ethnic groups.

The dried presumptive *E. coli* cultures from the 96-well assay plates were recovered at the WSU laboratory by adding 150 µL of LB broth into each well of the plates and incubating overnight at 37˚C. Duplicate working plates were prepared from the original plates; glycerol was added to the original plates and stored at -80˚C. The identity of n = 800 presumptive *E. coli* isolates was confirmed by their colony morphology on MacConkey agar (streaking those isolates on MAC plates) and by testing for the presence of *uidA* marker using a PCR assay [2]. As part of a related project, we later conducted whole-genome sequencing for 183 putative *E. coli* isolates from four Maasai households, of which 90·7% were *E. coli* and 7·7% were *Enterobacter cloacae* and for 25 putative *E. coli* isolates from waters, of which 92% were *E. coli* and 8% were *Enterobacter cloacae*.

2.3. Estimating the load (bacteria per ml) and isolation of lactose fermenting bacteria in milk

Milk samples transported to the NM-AIST laboratory where they were serially diluted (10-fold) by using sterile phosphate buffered saline; scrubbed swab samples were multiple times squeezed between fingers inside the Whirl-Paks. Milk samples (50 µl from 10^0 to 10^{-2} dilutions) and undiluted swab extracts were plated on to MacConkey agar plates by using sterile glass-beads and then incubated overnight at 37°C in a stationary incubator. After 24 h the plates were examined for the presence of presumptive *E. coli* and/or other lactose-fermenting bacteria. When tested in this manner, the abundance of *E. coli* was limited in the milk samples and hence, the number of Gram-negative lactose-fermenting colonies was enumerated. The bacterial load (log_{10}) was estimated per mL of milk. The undiluted milk and swab samples were stored at -80°C for future use. A related whole-genome sequencing project from four Maasai households indicated that milk isolates were composed of approximately 21·7% and 68·3% *E. coli* and *E. cloacae*, respectively, while swab isolates included 9·5% *E. coli*, 34·4% *E. cloacae* and 35·1% of *Klebsiella* sp.

2.4. Estimating the bioavailability of oxytetracycline residues in milk

To determine if single boiling events can affect the biological activity of oxytetracycline residues in milk, we used a competition experiment between a resistant [SSuT-6; (*22*)] and an isogenic susceptible *E. coli* strain (the resistance genes were deleted). Briefly, milk (100 mL, pasteurized, homogenized) was transferred into sterile conical flasks and then supplemented with oxytetracycline [0.5 µg/mL final concentration before being serially diluted (2-fold) to attain 0·25 and 0·125 µg/mL concentrations]. Aliquots (5 ml) from each dilution were transferred into 15-mL polystyrene tubes and the remaining milk was heated until boiling for approximately 2-3 min. Boiled milk was cooled to room temperature and 5 mL aliquots were prepared. Drug-resistant or susceptible *E. coli* culture (isogenic strains) were added to every aliquot to a final concentration of approximately 6 log_{10} cfu/mL after which the milk samples were incubated at 37°C for 24 h. After incubation, the colony forming units of resistant and susceptible *E. coli* were enumerated on MacConkey agar plate with and without oxytetracycline (16 µg/mL). The colony forming units were log_{10} transformed and competitive indices were calculated using the following formula (*23*). Competitive Index (CI) = (X-Y) / (X+Y), where X – number of resistant and Y – number of susceptible *E. coli* populations. Values >0 indicate that the resistant strain was numerically dominant while values <0 indicate that the susceptible strain dominated.

3. Data analyses and results

3.1. Risk factors for AR bacteria in people.

a. Lasso Models

The Lasso is a shrinkage and variable selection method. It minimizes the usual sum of squared errors bounding the sum of the absolute values of regression coefficients. Variable selection can be notoriously difficult for high dimensional data (the number of variables exceeds the sample size) or highly correlated variables with different magnitudes [3–5]. As a result, various regularization approaches have been widely used. In this case, the data are not high dimensional but some variables are highly correlated. To this end, we adopted the method originally proposed by Wang et. al. over a range of penalties [4], shown to be effective in the low dimensional setting [6].

Let N denote the number of isolates and P the number of variables. A number of bootstraps B and a range of penalty parameters Λ were chosen. We note that the choice of Λ is typically not influential on the results [4] and we chose the minimal value using a simple permutation test to ensure that any relationship between our variables and the response was stronger than if these relationships occurred by chance [7]. Our maximum penalty parameter was chosen such that at least one variable had a non-zero coefficient.

All continuous variables were standardized before all analyses. For each ethnic group separately, variables with a high proportions of missing values or with no variability were excluded before analysis. We chose a 5% cut

off threshold for missing data in order to retain as many variables as possible while minimizing exclusion of households.

The algorithm employed was as follows:

For each $\lambda \epsilon \Lambda$,

For the first bootstrap iteration (b = 1),

Step 1 Draw a random subsample of size [N/2] without replacement [4].

> **Step 1a** Randomly select a subset of q variables such that q < P. We set coefficient estimates of those variables not selected equal to 0.
>
> **Step 1b** For the selected q variables, obtain regression coefficients $\widehat{\beta_1^\lambda}$ using the R package glmmLasso [8].

For b = 2, …, B,

Step 2 Estimate the importance of each variable (p=1,…,P) [3] where importance is given by

$$I_{b,p}^{\lambda} = (b-1)^{-1} \sum_{r=1}^{b-1} \left| \widehat{\beta_{r,p}^{\lambda}} \right|.$$

> **Step 2a** Estimate selection probabilities of each variable, where the selection probability is given by

$$SP_{b,p}^{\lambda} = \frac{I_{b,p}^{\lambda}}{\sum_{p=1}^{P} I_{b,p}^{\lambda}}.$$

Step 3 Draw a random subsample of size [N/2] without replacement.

> **Step 3a** Select a subset of q variables such that q < P using selection probabilities $SP_{b,.}^{\lambda}$. The value of q was allowed to change at each bootstrap iteration. We set coefficient estimates of those variables not selected equal to 0.
>
> **Step 3b** For the selected q variables, obtain penalised regression coefficients $\widehat{\beta_b^\lambda}$.

Step 4 After B bootstraps, use a parametric test for variable selection (PSTVSboot) adopted by Park et al (*5*) to formally select variables. We set if variable p was included using regularisation λ and if variable p was not included using regularisation λ.

Step 5 For each p=1,…,P, inclusion probabilities were estimated as

$$IP_p = \frac{\sum_{\lambda \epsilon \Lambda} D_p^{\lambda}}{|\Lambda|}.$$

b. Variables entered into Lasso Models

A list of the 56 variables initially entered into each Lasso model is given in Table 3. Results of lasso selection are provided for all groups combined (Fig. 1a) and for each ethnic group separately (Fig1b-Fig1d). Odds ratios (OR) for the best fit models (assessed using the Akaike information criterion) are reported in Table 4a-Table 4c.

Table S2. Descriptions of the 56 variables used in the random Lasso regression models.

**Variable name**	**Variable description**
abruse_scale	A scale of antimicrobial use (see "Scaling Antimicrobial Use" below for further description)
abruse_comp1	Component 1 of PCA (see "PCA of Antimicrobial Use" below for further description)
abruse_comp2	Component 2 of PCA (see "PCA of Antimicrobial Use" below for further description)
boil_milk	A dichotomous variable indicating whether the household reported boiling milk
prof_modern	A dichotomous variable indicating whether the household purchased biomedical livestock medicine (antimicrobials) from a professional veterinary service.
prof_vacc	A dichotomous variable indicating whether the household purchased vaccines from a professional veterinary service.
child_mort	Whether the household had any children that didn't survive until one years of age
children_under_5_vaccinated	A dichotomous variable indicating whether children under 5 within the household received vaccinations
communal_graze	A dichotomous variable indicating whether the household had access to communal grazing lands
milk_consume_sick	A dichotomous variable indicating whether the household reporting consuming milk from sick cows.
disease_of_herd	A dichotomous variable indicating whether a household eats animals that die (1) or do not eat (0), which includes skin and bury, bury without skinning, burn, or cook for dogs and pigs.
purchase_distance	A dichotomous variables indicating whether a household reported that disease factors limited them from increasing their herd size.

village_distance	distance from household to the nearest village in km
eat_meat_weekly	A dichotomous variable indicating whether the household ate meat (either beef, goat, chicken, or pig) 3 or more times a week (coded 1) or less than three times a week (coded 2)
education	Education level was recorded for the household head and included none, some primary, completed primary, some high school, completed high school, some college, completed college, and postgraduate and above.
ethnic	Ethnic group include Maasai (1), Arusha (2), and Chagga (3)
recent_illness_num	The number of livestock who were sick during the most recent disease outbreak
antimicrobials_present	A dichotomous variable indicating whether the household had veterinary antimicrobials on the premises.
livestock_contact	Whether livestock came in contact with livestock from other households when grazing or taking water.
water_sharing_animals	Whether water sources were shared with livestock and wildlife or were for people only
market_number	The number of markets used to sell or buy livestock
milk_consume	Liters of milk consumed by the household. In the Maasai consumption was reported at the boma level (multiple household units) so liters were divided by number of households.
min_to_health_center_foot	The number of minutes it takes a healthy person to walk to the nearest health clinic/hospital.
move_livestock_normal	Number of times a household moved their livestock in a normal year. Options included 0-6 and NA for households that did not have livestock.
livestock_distance_normal	Distance livestock are moved in a normal year (e.g. to a temporary boms). Options include: less than one day walk, 1-2 days, 3-4 days, more than 4 days and N/A.

no_boil_milk_consumption	Liters of milk consumed in household that reported not boiling milk
dead_calves	The number of calves born dead in the last year as reported by the household
cattle_managed	Reported number of cattle the household was managing for someone else.
health_care_visits	Number of reported times any household member visited a health clinic or hospital in the last three months. Options were: 0-1 time, 2-4 times. 5-10 times, 11-20 times, Over 20 times.
kids_dead	Reported number of kids born dead in the last year
cattle_purchased	Reported number of cattle the household purchased last year
shoats_purchased	Reported number of sheep and goats the household purchased last year.
livestock_purcahsed	Reported number of cattle, sheep and goats the household purchased last year
livestock_exchange	Reported number of people that the household exchanged livestock with in the last year, this could include livestock traders, family, friends, and in-laws (bride-wealth payment)
shoats_managed	Reported number of sheep and goats the household was managing for someone else.
livestock_managed	Reported number of cattle, sheep and goat the household was managing for someone else.
prof_serv	Whether the household reported using a professional veterinary service in the last year,including veterinarian and livestock officer
season	Season of data collection. The long rainy season is normally from beginning of March to end of May. Tong dry season is normally from the beginning of June to the end of November

disease_avoid	Self-reported number of steps taken to avoid livestock disease. These steps include: (1) Keep calves separate (2) Make a shed or pen to prevent contact from other domestic and wild animals, (3) Keep new cattle separately, (4) Graze sick cattle separately, (5) Feed supplementation (6) Buy new cattle from local area (7) Vaccination, (8) Seek professional veterinary services, (9) Use traditional treatment, and (10) spraying for disease.
sickness_interrupt	Self-report response to "has sickness in your herd reduced the time available for other business/work in the last year
toilettype	Toilet type was a dichotomous variables indicating whether the households had a toilet(1) or not (0). Pit, trench and flush toilets were coded as toilets. Bathroom locations that were in the bush, even if in a dedicated location, were coded as Bush (0)
livestock_away	The total number of livestock that are kept away from the bomas/household for extended periods of time. Livestock kept away include cattle, sheep, and goats. In the Maasai, for example, livestock are often kept at temporary bomas many kilometers<br>away from the boma.
livestock_home	The total number of livestock that leave the boma/household in the morning and return at night after grazing. Livestock includes cattle, sheep, and goats.
livestock_inout	The total number of livestock that stay in the boma/household all day. Livestock includes cattle, sheep, and goats.
total_birds	The total number of chickens owned by the household, this includes cocks and hens
dogs_number	The total number of dogs that the household owned.
urban	Composite of whether the household had electricity (mainline or solar) and the number of items the household owned indicative of a more urban lifestyle, including radio, television, refrigerator, motorcycle/scooter, car truck and cell phones
use_antimicrobial_illness_1_human	A dichotomous variable indicating whether the most recent exotic medicine reported was an antimicrobial or non-antimicrobial.
vet_seek	Self-report response to "Do you consult a veterinarian or other livestock care professional when your animals are sick?" Coded 1 Yes 0 = NO

vaccine	Self-report response on the number of different vaccinations (e.g. foot and mouth disease, East Coast Fever)
waterhole_number	Reported number of water holes used by household for livestock.
Share_water	Whether the household reported water sources that were shared (communal well, lake/ river water/seasonal stream) or not shared (stand pipe/private well/pay for delivery/neighbor stand pipe).
water_treatment	Whether the household reported treating their drinking water. Treatments included boiling and chemical treatments.
withdrawal	Whether the household reported observing withdrawal from antimicrobials. Options were using products after: the animal was healthy, 10 days after treatment, 3 days after treatment, and immediately after. Immediately was coded as not observing withdrawal (0) and all other options were recoded as observing withdrawal.
village_distance	Distance from households to the nearest village
total_dead	Due to low livestock sizes in Chagga households, we combined dead_calves and kids_dead additively to create a variables which gives the reported total number of kids and calves born dead.

c. Scaling Veterinary Antimicrobial Use

We developed a scale of veterinary antibiotic use by combining self-report items and direct observation of antibiotics on-hand in the household (see Table 4). Self-reported indicators of lay veterinary antibiotic use included self-reported use, and ownership of syringes and needles for intramuscular or subcutaneous injections. In addition, we observed and inventoried antibiotics on hand in the household. We assumed that the combination of self-reported and direct observation items offers a robust scale with good sensitivity and specificity for veterinary antibiotic use, resulting in less complicated interpretation of *P*-values in regression models [9].

We used a mixed data factor analysis [10] to explore the properties of scale. The first unrotated component was a weighted average of keeping needles and syringes and using veterinary antibiotics (abruse_comp1, Fig. S3, Table S3) and accounted for 66% of the variance. We interpreted abr_use_comp1 as households that administered livestock antibiotics in the most recent illness. The number of veterinary antibiotics kept at hand dominated the second unrotated component (abr_use_comp2) and so indicated high levels of antimicrobial usage and accounted for 23% of variation. Component 3 (ab_ruse_comp3) accounted for 11% of that variation and indicated households that used antibiotics in the most recent livestock illness but didn't keep needles and syringes (as so perhaps were less likely to self-administer).

Table S3. Ethnic-group comparison of loadings from factor analysis of livestock antimicrobial use composed of the number of veterinary antimicrobials kept at home (continuous), the use of antimicrobials in a recent livestock illness (yes or no) and keeping needles and syringes (yes or no).

Variable	abruse (component 1)	abruse2 (component 2)	abruse3 (component 3)
Number of VAs kept**	0.68	0.73	0.08
Use VAs (No)*	-1.26	0.33	-0.22
Use VAs (Yes)*	1.13	-0.30	0.20
Keep needle and syringe (No) *	-1.14	0.16	0.21
Keep needle and syringe (Yes) *	1.37	-0.19	-0.25

*self-report; **direct observation

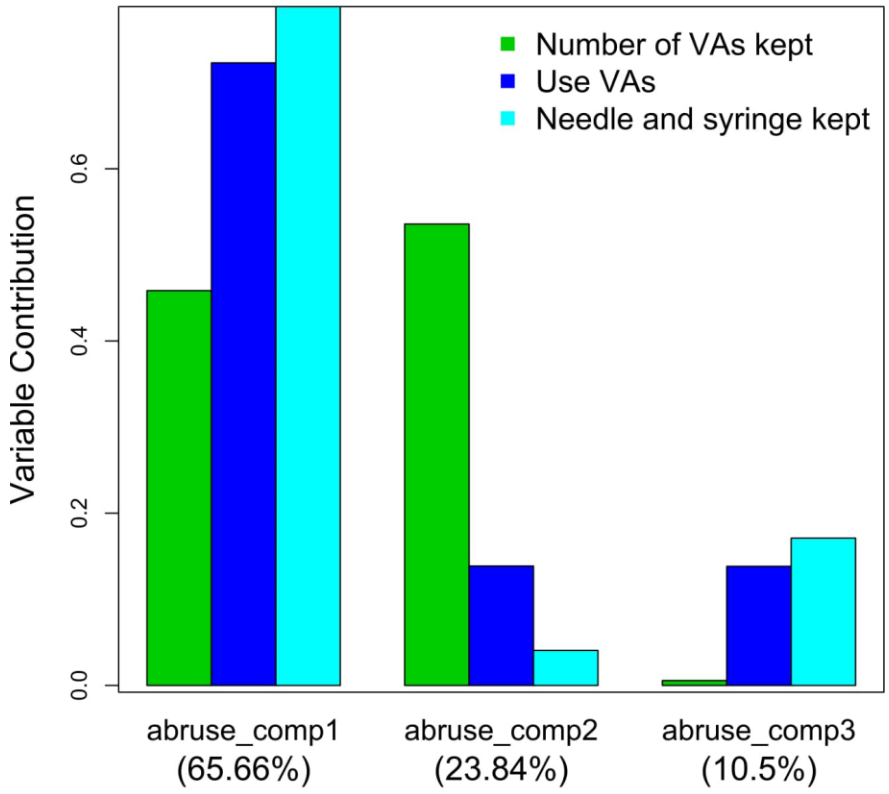

Figure S3. Mixed data factor analysis of veterinary antimicrobial usage in terms of number of veterinary antimicrobials [VAs] kept at the household, whether VAs were used and whether needles and syringes were kept. The percentage of variance explained by each component is shown in x-axis parentheses.

3.2 Results of Lasso analyses pooled across groups and for each ethnic group.

Following standard approaches, variables with $IP_p > 0 \cdot 6$ (Figs. S4a-S4d) [4] were used in an unpenalised regression to obtain reliable and interpretable regression coefficients (Tables S4a-S4d). For each antimicrobial, we set B=500 and present models including all variables with $IP_p > 0 \cdot 6$(Lasso variables selection) and models with lowest AIC values (Best fit model selected via AIC minmization). The threshold value of 0·6 was chosen within a range of values suggested by Meinshausen [4]. We note the relationship between the expected number of falsely selected variables and the threshold value but our results showed little variability for threshold values greater than 0·6, as expected [4].

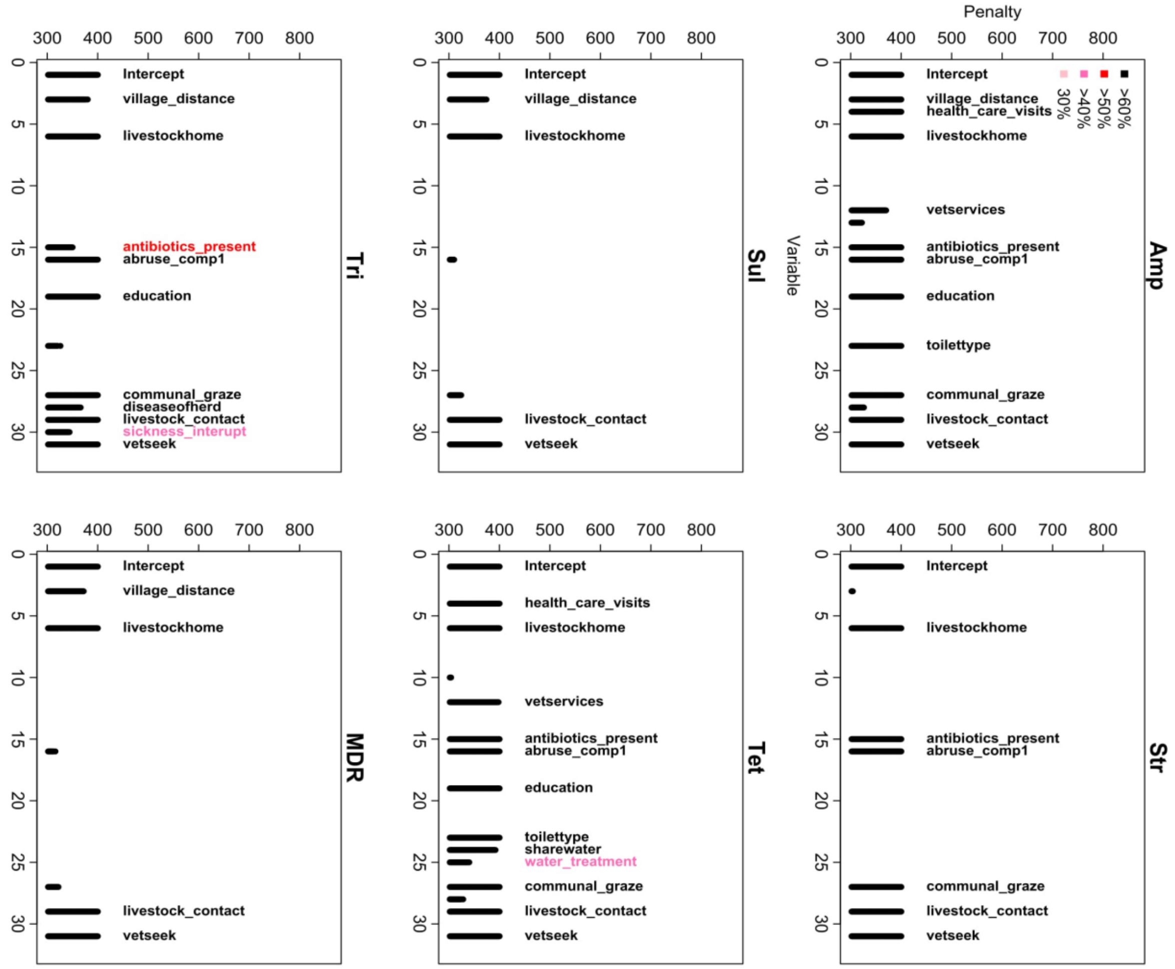

Figure S4a. Pooled ethnic group (Arusha, Chagga, Maasai) risk factors selected by Lasso models for (Amp)icillin, (Tet)tracycline, (Tri)methoprime, (Sul)famethoxazole, (Str)eptomycin and multidrug resistant (MDR) phenotypes. Variables selected more than 60% (black), 50% (red), 40% (dark pink) and 30% (light pink) of the time in random Lasso (section S3) from full set of variables (Table S4). The x-axis is the penalty parameter (see Section 3.1 above) and the y-axis is the number scales representing the variables in the model.

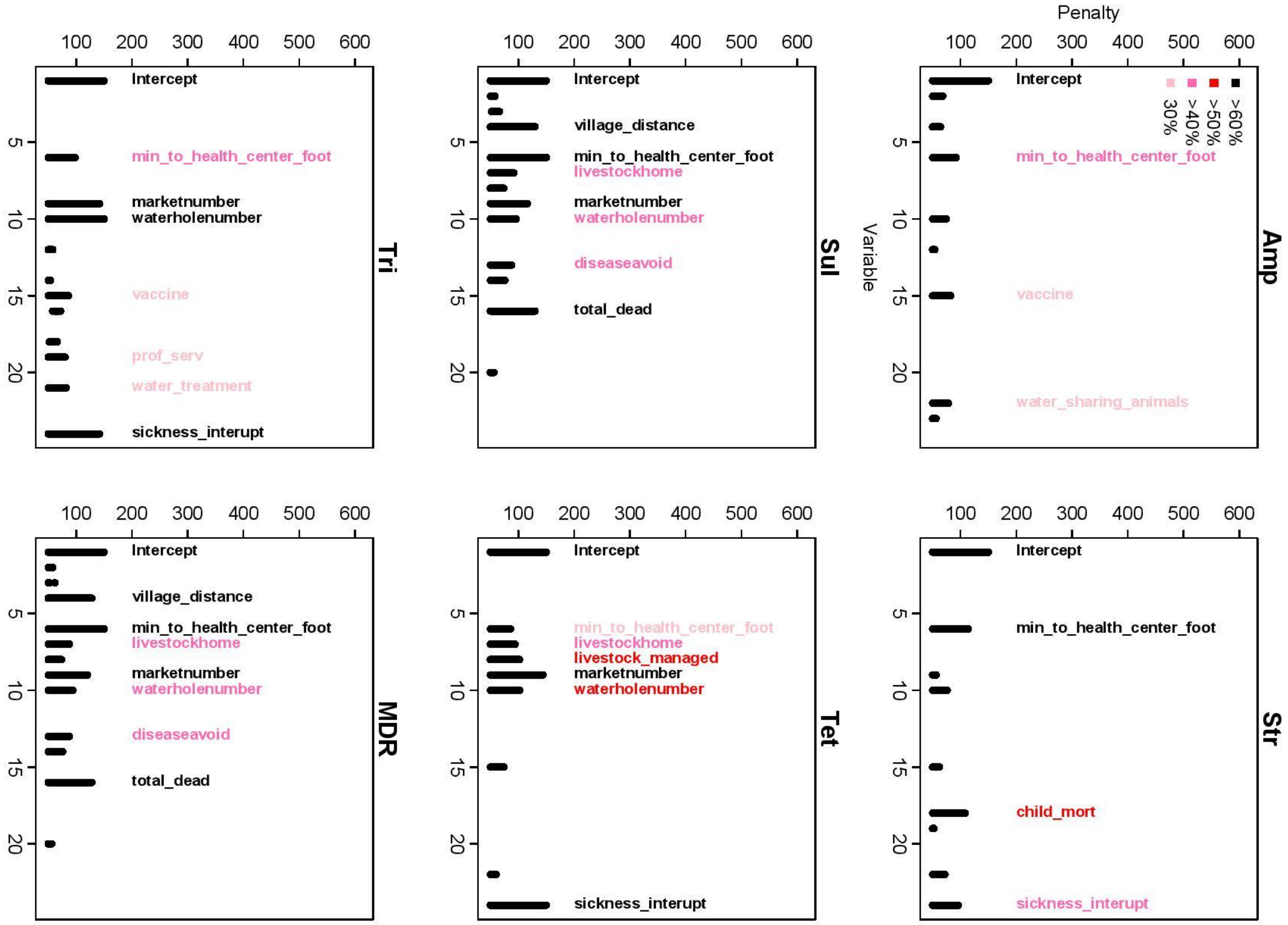

Figure S4b. Chagga ethnic group risk factors selected by Lasso models for (Amp)icillin, (Tet)tracycline, (Tri)methoprime, (Sul)famethoxazole, (Str)eptomycin and multidrug resistant (MDR) phenotypes. Variables selected more than 60% (black), 50% (red), 40% (dark pink) and 30% (light pink) of the time in random Lasso (section S3) from full set of variables (Table S2). The x-axis is the penalty parameter (see Section 3.1 above) and the y-axis is the number scales representing the variables in the model.

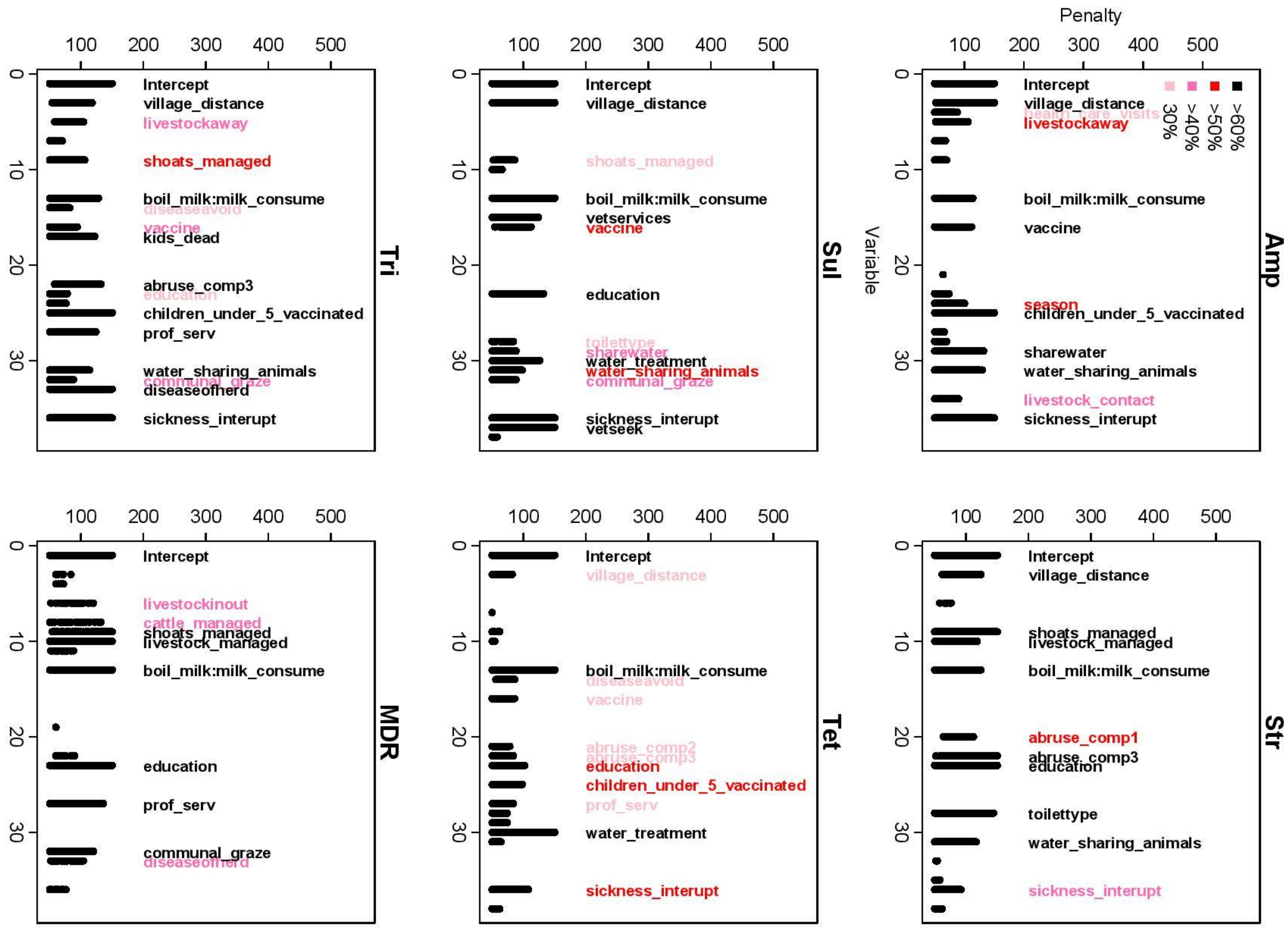

Figure S4c. Maasai ethnic group risk factors selected by Lasso models for (Amp)icillin, (Tet)tracycline, (Tri)methoprime, (Sul)famethoxazole, (Str)eptomycin and multidrug resistant (MDR) phenotypes. Variables selected more than 60% (black), 50% (red), 40% (dark pink) and 30% (light pink) of the time in random Lasso (section S3) from full set of variables (Table S2). The x-axis is the penalty parameter (see Section 3.1 above) and the y-axis is the number scales representing the variables in the model.

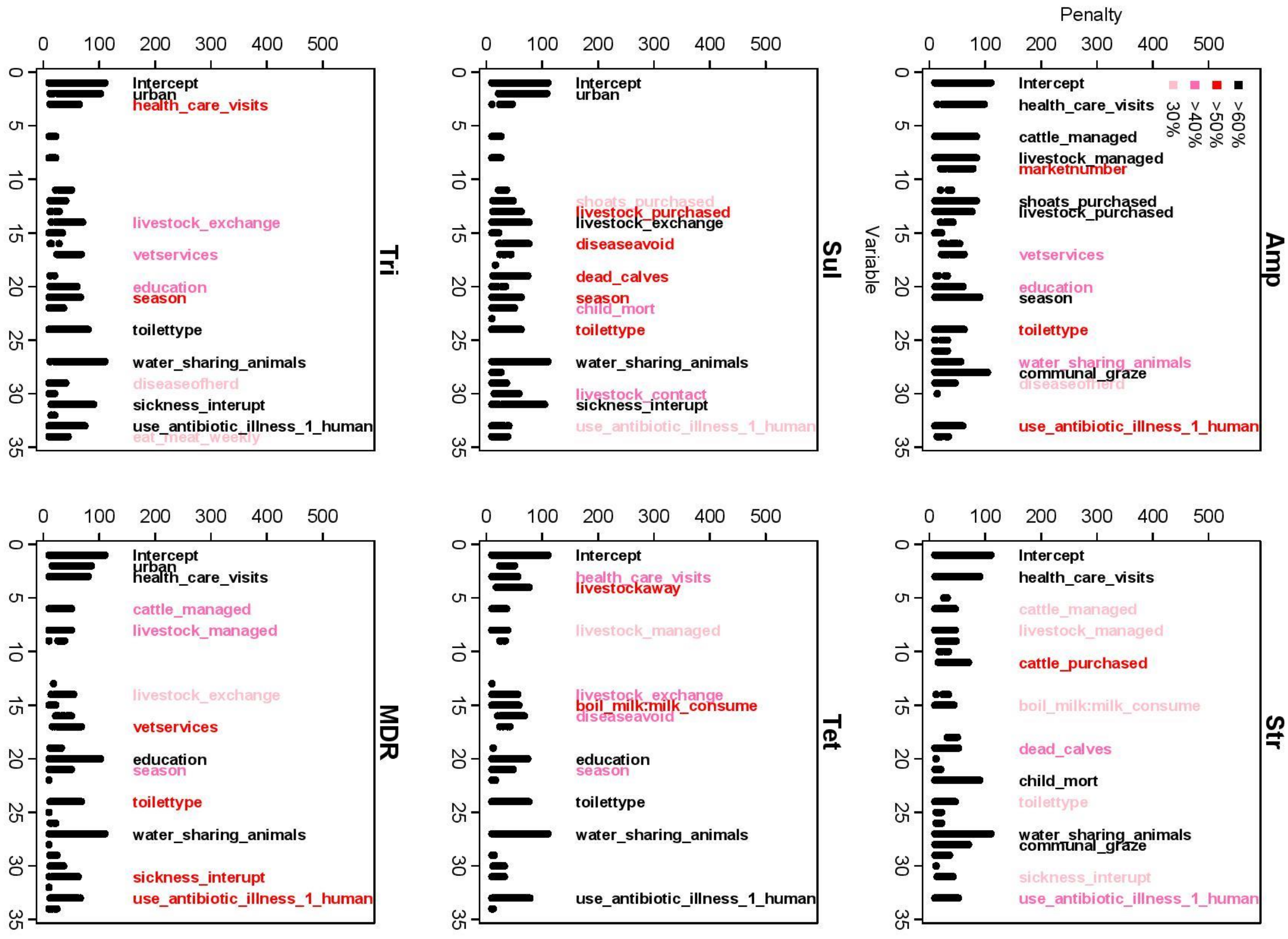

Figure S4d. Arusha ethnic group risk factors selected by Lasso models for (Amp)icillin, (Tet)tracycline, (Tri)methoprime, (Sul)famethoxazole, (Str)eptomycin and multidrug resistant (MDR) phenotypes. Variables selected more that 60% (black), 50% (red), 40% (dark pink) and 30% (light pink) of the time in random Lasso (section S3) from full set of variables (Table S2). The x-axis is the penalty parameter (see Section 3.1 above) and the y-axis is the a number scales representing the variables in the model.

Table S4a. Risk factors for antimicrobial-resistant bacteria from Chagga households. Mixed model results for Chagga households using full set of variables included more than 60% in random Lasso (Fig S3b) clustered at the household level (Lasso variable selection). Models that lower AIC amongst all possible combinations of Lasso selected variables (Best fit model selected via AIC minimization). '-' represents variables not present in model with lowest AIC.

**Antimicrobial**	**Variable**	**Proportion of time included in final lasso model**	**Lasso variable selection**		**Best fit model selected via AIC minimization**	
			Estimated odds	*P*	Estimated odds	*P*
**Ampicillin**	intercept	1	0.14	<0.0001	-	-
**Streptomycin**	intercept	1	0.12	<0.0001	0.12	<0.0001
	mins_to_health_center	0.647	0.68	0.1240	0.68	0.1240
**Sulfamethoxazole**	intercept	1	0.34	0.0002	0.35	<0.0001
	village_distance	0.78	1.11	0.7163		
	mins_to_health_center	1	0.75	0.2750	0.65	0.0619
	market_number	0.67	0.99	0.9791	-	-
	total_dead	0.80	0.69	0.2024	-	-
**Tetracycline**	intercept	1	0.37	0.0011	0.37	0.0011
	market_number	0.941	0.68	0.0646	0.68	0.0646
	sickness_interrupt	1	0.34	0.0086	0.34	0.0086
**Trimethoprim**	intercept	1	0.26	0.0003	0.19	<0.00

**e**						01
	market_number	0.922	0.76	0.2784	-	-
	waterhole_number	1	1.65	0.0333	1.89	0.0053
	sickness_interrupt	0.922	0.57	0.2551	-	-
**Multidrug Resistant**	intercept	1	0.17	<0.0001	0.17	<0.0001
	village_distance	0.784	1.07	0.8290	-	-
	mins_to_health_center	1	0.86	0.6030	-	-
	market_number	0.796	0.78	0.4230	0.70	0.1330
	total_dead	0.784	0.91	0.7630	-	-

Table S4b. Risk factors for antimicrobial-resistant bacteria from Arusha households. Mixed model results for Arusha households using full set of variables included more than 60% in random Lasso (Fig S3c) clustered at the household level (Lasso variable selection). Models that lower AIC amongst all possible combinations of Lasso selected variables (Best fit model selected via AIC minimization). '-' represents variables not present in model with lowest AIC.

Antimicrobial	Variable	Proportion of time included in final lasso model	Lasso variable selection		Best fit mode[illegible] selected via AI[illegible] minimization[illegible]
			Estimated odds	*P*	Estimated odds
**Ampicillin**	intercept	1	4.97	<0.0001	5.00
	health_care_visits	0.90	1.51	0.1992	-
	cattle_managed	0.71	NA	NA	-
	livestock_managed	0.71	1.12	0.7614	-
	shoats_purchased	0.67	0.07	0.1146	0.05
	livestock_purchased	0.61	10.73	0.1751	16.12
	season	0.80	0.03	0.0121	0.02
	communal_graze	0.92	0.16	0.0222	0.18
**Streptomycin**	intercept	1	0.10	0.0002	0.09
	health_care_visits	0.86	1.97	0.0394	1.98
	child_mortality	0.82	0.77	0.8373	-
	water_sharing_animals	1	11.59	0.0094	11.70
	communal_graze	0.61	0.16	0.0438	0.16
**Sulfamethoxazole**	intercept	1	0.33	0.0589	0.27
	urban	0.90	0.70	0.1214	0.68
	livestock_exchange	0.69	0.59	0.0586	0.56
	dead_calves	0.61	1.14	0.5510	-

	water_sharing_animals	1	8.76	<0.0001	9.23
	sickness_interrupt	0.90	0.76	0.6040	-
**Tetracycline**	intercept	1	0.09	0.0334	0.07
	livestock_away	0.61	0.79	0.1713	0.77
	education	0.63	0.65	0.4159	-
	toilet_type	0.71	3.93	0.1711	4.10
	water_sharing_animals	1	6.18	0.0011	6.23
	use_antimicrobial_illness_1_human	0.67	0.19	0.1283	0.16
**Trimethoprime**	intercept	1	0.24	0.2496	0.57
	urban	0.75	0.74	0.3111	0.65
	toilet_type	0.71	5.35	0.1474	-
	water_sharing_animals	1	2.42	0.1462	3.47
	sickness_interrupt	0.82	0.54	0.2830	-
	use_antimicrobial_illness_1_human	0.65	0.47	0.5429	-
**Multidrug Resistant**	intercept	1	0.32	0.0577	0.35
	urban	0.75	0.79	0.3915	-
	health_care_visits	0.69	1.42	0.1816	1.47
	education	0.92	0.49	0.2364	0.9
	water_sharing_animals	0.98	7.25	0.0006	7.54

Table S4c. Risk factors for antimicrobial-resistant bacteria from Maasai households. Mixed model results for Maasai households using full set of variables included more than 60% in random Lasso (Fig Sd) clustered at the household level (Lasso variable selection). Models that lower AIC amongst all possible combinations of Lasso selected variables (Best fit model selected via AIC minimization). '-' represents variables not present in model with lowest AIC.

**Antibiotic**	**Variable**	**Proportion of time included in final lasso model**	**LASSO variable selection**		**Best fit model selected via AIC minimization**	
			Estimated odds	*P*	Estimated odds	*P*
**Ampicillin**	Intercept	1	0.304	0.5191	0.304	0.5192
	village_distance	0.98	0.81	0.3856	-	-
	boil_milk	0.65	4.86	0.0320	4.90	0.0322
	boil_milk_consumption	0.65	0.32	0.0342	0.31	0.0268
	milk_consume	0.65	2.63	0.0213	2.74	0.0169
	children_under 5_vaccinated	1	7.83	0.0168	7.24	0.0219
	share_water	0.82	0.17	0.0718	0.17	0.0727
	sickness_interrupt	0.98	0.42	0.1758	0.37	0.1170
	vaccine	0.63	0.71	0.1670	0.67	0.0868
	water_sharing_animals	0.78	7.21	0.1181	6.30	0.1470
**Tetracycline**	intercept	1	0.49	0.1460	0.45	0.0911
	boil_milk	1	13.90	0.0003	14.88	0.0002
	boil_milk_consumption	1	0.18	0.0009	0.16	0.0004
	milk_consume	1	3.00	0.0079	3.10	0.0058

	water_treatment	1	0.59	0.4594	-	-
**Trimethroprime**	intercept	1	0.13	0.0781	0.13	0.0969
	village_distance	0.67	0.96	0.8561	-	-
	boil_milk	0.78	10.16	0.0022	10.70	0.0018
	boil_milk_consumption	0.78	0.25	0.0167	0.21	0.0044
	milk_consume	0.78	2.44	0.0613	2.89	0.0157
	children_under_5_vaccinated	1	5.60	0.0645	5.53	0.0632
	disease_of _herd	1	2.23	0.1031	2.19	0.1126
	kids_dead	0.73	1.26	0.4122	-	-
	sickness_interrupt	1	0.31	0.0876	0.31	0.0755
**Sulfamethoxazole**	intercept	1	1.09	0.9142	0.41	0.2248
	village_distance	1	0.66	0.1110	0.61	0.0597
	boil_milk	1	31.83	0.0001	32.46	<0.0001
	boil_milk_consumption	1	0.12	0.0008	0.12	0.0002
	milk_consume	1	4.09	0.0038	3.97	0.0023
	education	0.82	0.39	0.0634	2.08	0.1348
	sickness_interrupt	1	0.20	0.0166	0.21	0.0198
	vet_services	0.71	0.55	0.0136	0.56	0.0147
	water_treatment	0.75	0.37	0.2238	-	-
**Streptomycin**	intercept	1	0.83	0.8885	0.17	0.0003

	village_distance	0.63	0.87	0.5497	-	-
	boil_milk	0.75	7.76	0.0067	8.76	0.0043
	boil_milk_consumption	0.75	0.27	0.0148	0.23	0.0073
	milk_consume	0.75	2.98	0.0147	3.39	0.0056
	abruse3	0.94	0.68	0.2379	-	-
	shoats_managed	1	2.04	0.0460	2.09	0.0570
	toilet_type	0.94	2.18	0.2782	3.13	0.1039
	water_sharing_animals	0.67	0.24	0.2477	-	-
**Multidrug Resistant**	intercept	1	0.47	0.5842	0.25	0.0038
	boil_milk	1	16.53	0.0001	15.49	0.0001
	boil_milk_consumption	1	0.19	0.0029	0.21	0.0021
	milk_consume	1	3.18	0.0112	2.92	0.0089
	education	1	1.75	0.2118	1.97	0.1129
	shoats_managed	0.76	3.53	0.2148	1.53	0.1101
	livestock_managed	0.9	0.40	0.3947	-	-
	prof_serv	0.76	0.99	0.9921	-	-
	communal_graze	0.71	0.52	0.6400	-	-

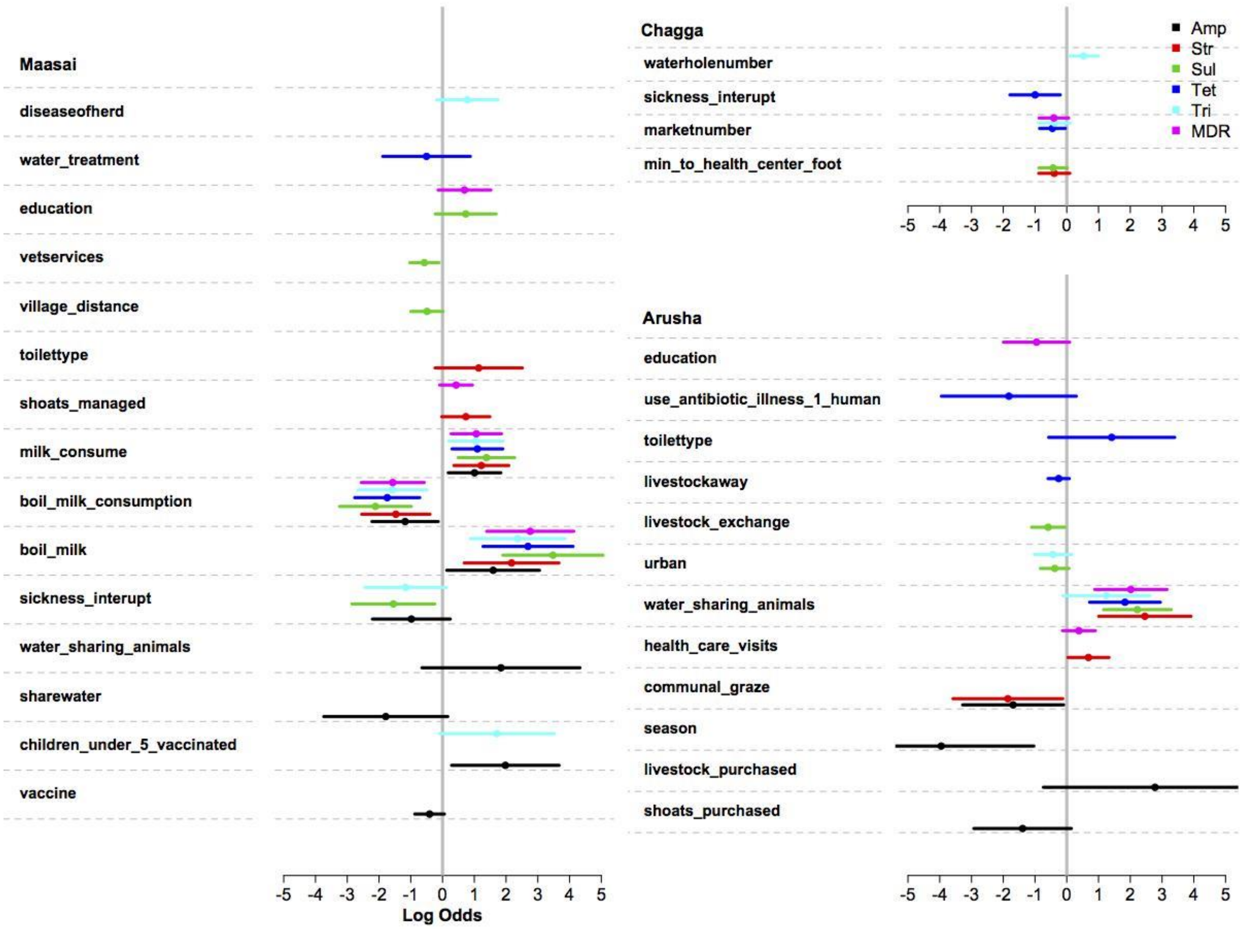

Figure S5. Risk factors for antimicrobial resistant *E. coli* isolated from stool samples of pooled across Maasai, Arusha, and Chagga people. Estimated log odds shown as points and 95% confidence interval shown in bars. Colors indicate (Amp)icillin, (Str)eptomycin, (Sul)famethoxazole, (Tet)tracycline, (Tri)methoprime, or MDR (multidrug resistant phenotype). See Table 2 for variable descriptions.

4. Analysis of bacterial load in milk and swabs of milking containers

There were no significant differences in the bacterial load (colony forming units per ml; cfu/ml) across ethnic groups ($F_{(1,165)}$ = 2·9, P = 0·09; Fig. 3B). Significant ($F_{(3,106)}$ = 6·06, P = 0·0008) differences were noted for different containers. A Tukey post-hoc test indicated these differences were driven by the carriage of lower (1·45 Log_{10} cfu/ml) loads in milk samples collected directly from an udder compared to samples collected from a calabash (3·13 Log_{10} cfu/ml), metal (3·60 Log_{10} cfu/ml), and or plastic containers (3·86 Log_{10} cfu/ml).

4.1 Analyses of the prevalence of antimicrobial resistant lactose-fermenting Gram-negative bacteria in milk and swab samples

A total of 8,106 lactose-fermenting bacteria from milk (n = 5,131) and swabs (n = 2,975) were characterized for their antimicrobial resistance patterns. Overall, these isolates expressed similar resistance patterns as fecal resistant bacteria *E. coli* (Fig. 1, main manuscript) with the highest resistance for Amp, Tri, Sul, Tet and Str (Fig. 3A, main manuscript). Interestingly, Maasai milk and swab isolates harbored a higher prevalence of

ciprofloxacin resistance (31·41% and 11·20%, respectively) compared to fecal isolates (Fig. 1, main manuscript) and Arusha milk isolates. Results from single-factor multivariate analysis of variance (MANOVA) shows that the Maasai milk harbored significantly higher levels of Sul (P = 0·003), Tet (P = 0·03) and Tri (P = 0·0007) resistant bacteria compared to the Arusha households (Fig. 3B). Except for Cip resistance, swab isolates from Maasai households (Fig. S5) and fecal isolates from Arusha and Maasai households (Fig. 1, main manuscript) showed similar resistance patterns. In addition, regression models comparing the average proportions of each antimicrobial-resistant lactose-fermenting bacteria also showed a positive correlation between milk and fecal patterns (Fig. S7).

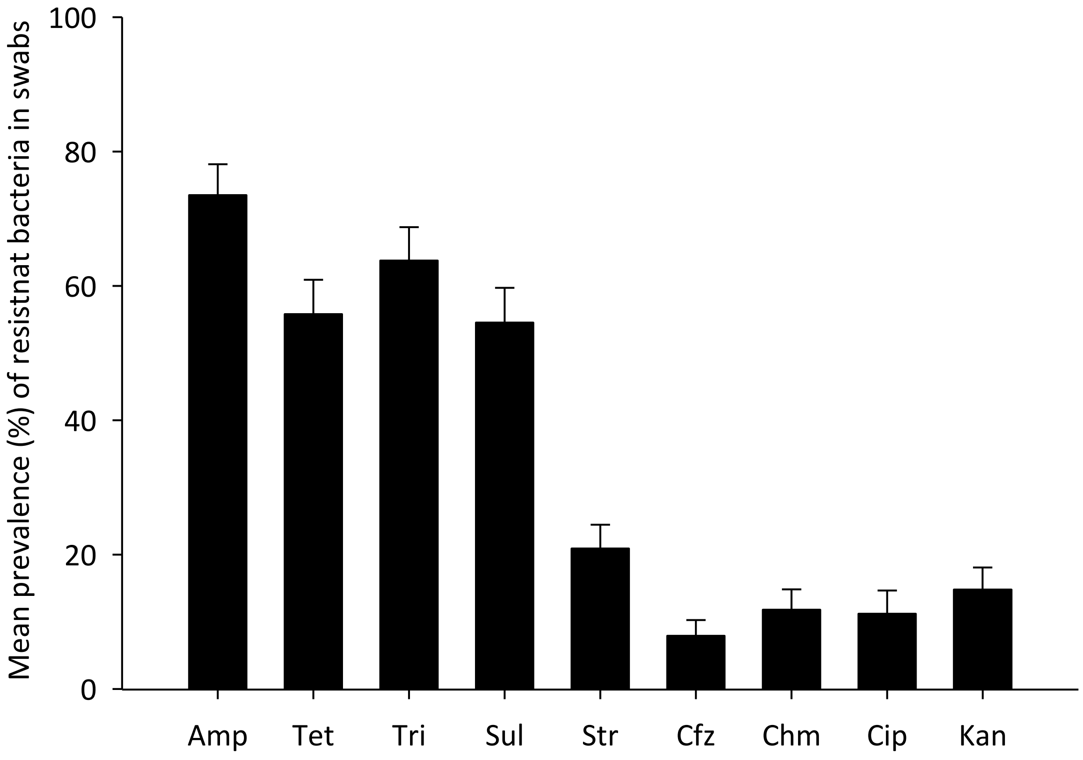

Figure S6. Mean prevalence of antimicrobial-resistant, Gram-negative lactose-fermenting bacteria (N=46) isolated from swabs of dry milk containers. Error bars are standard errors. The antimicrobial-resistance patterns of bacteria isolated from Maasai milk-containers are similar (except for Cip resistance) to bacteria isolated from Maasai milk samples. Milk containers were dry at the time of sampling. Antimicrobials include (Amp)icillin, (Tet)tracycline, (Tri)methoprime, (Sul)famethoxazole, (Str)eptomycin, Ceftazidime (Cfz), Chloramphenicol (Chm), (Cip)rofloxacin and (Kan)amycin. Error bars represent standard error.

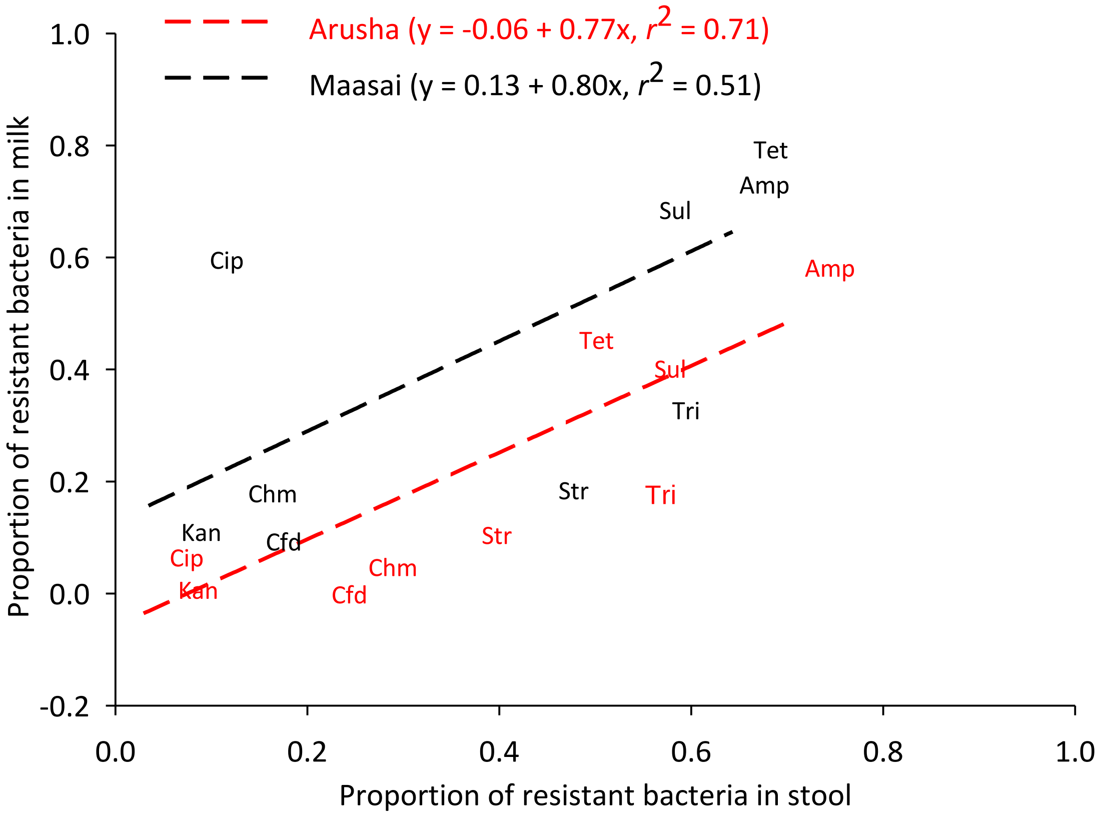

Figure S7. The proportion of Gram-negative lactose-fermenting bacteria resistant to nine antimicrobials for stool (x-axis) and milk samples (y-axis) collected from Maasai (in black; stool, n = 80; milk, n = 63) and Arusha (in red; stool, n = 55; milk, n = 31) households. These data are not paired at the household level and the samples were collected on different dates. For paired samples see Fig. 4a, in main manuscript.

4.2 Oxytetracycline residues remained biologically active after boiling milk.

We examined the biological activity of oxytetracycline after milk is boiled by using a competitive growth study between antibiotic-resistant and an isogenic susceptible *E. coli* populations. Our findings showed that the resistant *E. coli* population had an advantage over susceptible population in the presence of oxytetracycline, and that boiling milk had no significant effect on oxytetracycline (P = 0·64, Fig. S8).

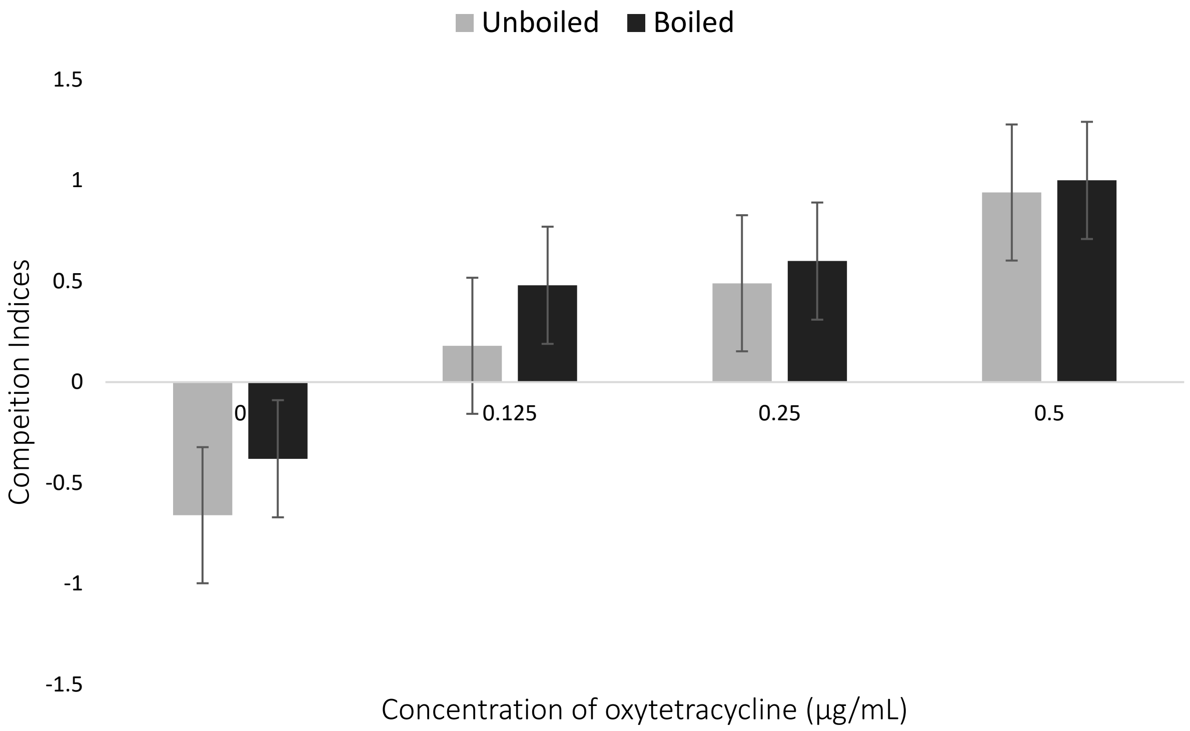

Figure S8. The mean competition index obtained from co-culture of a wild-type strain of *E. coli* (SSuT-6; (*25*)**) and an isogenic strain (lacking streptomycin, sulfadiazine and tetracycline resistance genes) in milk (n = 4 independent replicates).** Strong selection for the resistant population (values > 0) was evident in the presence of oxytetracycline in unboiled milk and after boiling milk samples that contained oxytetracycline. A two-factor ANOVA confirmed no differences between boiled and unboiled treatments ($P = 0.64$) while concentration was significant ($P < 0.001$; interactions $P = 0.99$). Error bars represent standard errors.

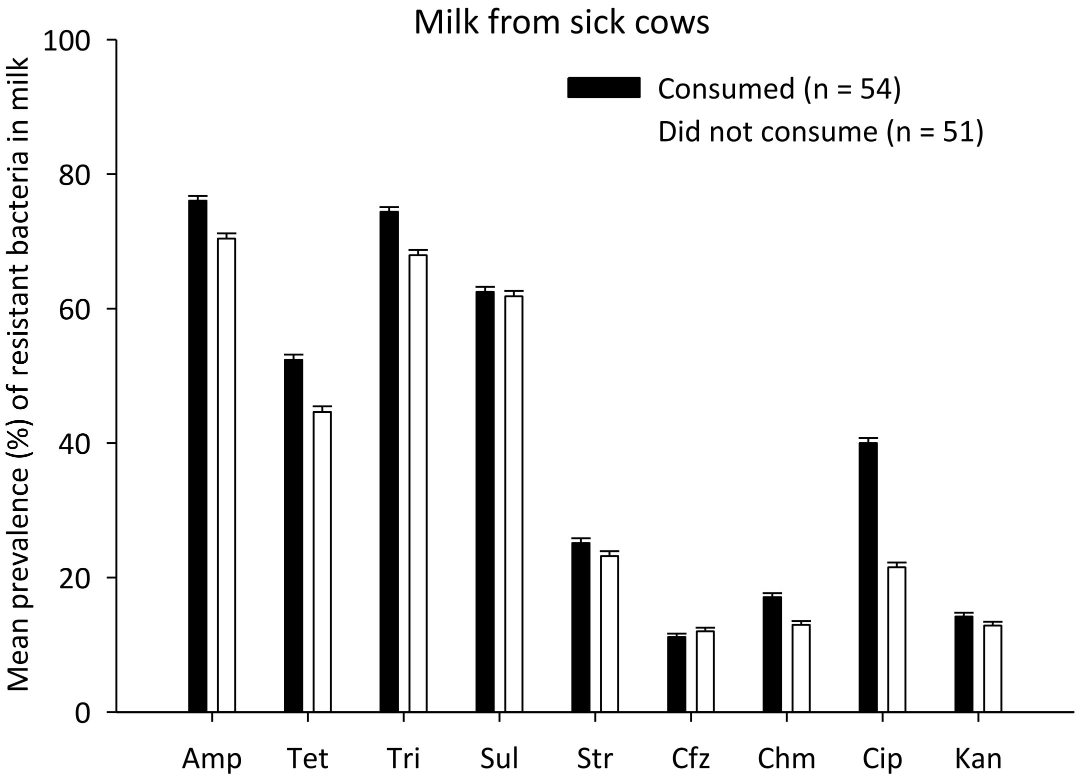

Figure S9. Mean prevalence of antimicrobial-resistant, Gram-negative lactose-fermenting bacteria (N = 6,458) isolated from milk samples (N=179) at Maasai and Arusha households. Results are summarized for households that consumed (filled bars) or that did not consume (open bars) milk from sick cows. Households that consume milk from sick cows had a significantly higher prevalence of antimicrobial-resistant bacteria (MANOVA, $P < 0.01$) for all antimicrobials tested except for Cfz, Kan and Sul. Antimicrobials include (Amp)icillin, (Tet)tracycline, (Tri)methoprime, (Sul)famethoxazole, (Str)eptomycin, Ceftazidime (Cfz), (Chl)oramphenicol, (Cip)rofloxacin and (Kan)amycin. Error bars represent standard error.

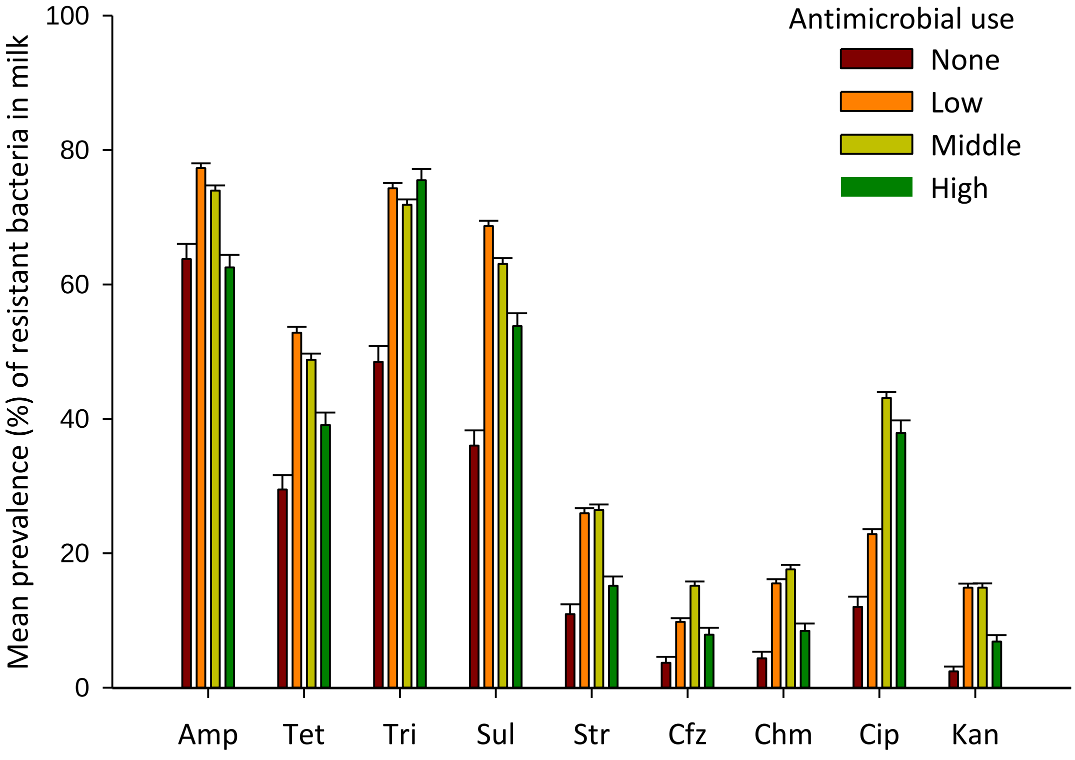

Figure S10. Mean prevalence of antimicrobial-resistant, Gram-negative lactose-fermenting bacteria (N = 7,505) isolated from milk samples (N=182) across Maasai and Arusha households with None (N = 21), Low (N = 27), Middle (N = 34) and High (N = 24) "levels" of reported veterinary antimicrobial use. Low, middle and high categories designate households that kept 1-2, 3-4, or 5-6 antimicrobials on hand. Except None, all households had syringes to administer antimicrobials to livestock. Antimicrobials include (Amp)icillin, (Tet)tracycline, (Tri)methoprime, (Sul)famethoxazole, (Str)eptomycin, Ceftazidime (Cfz), Chloramphenicol (Chm), (Cip)rofloxacin and (Kan)amycin. Significant differences between groups are discussed in the "Results" section. Error bars represent standard error.

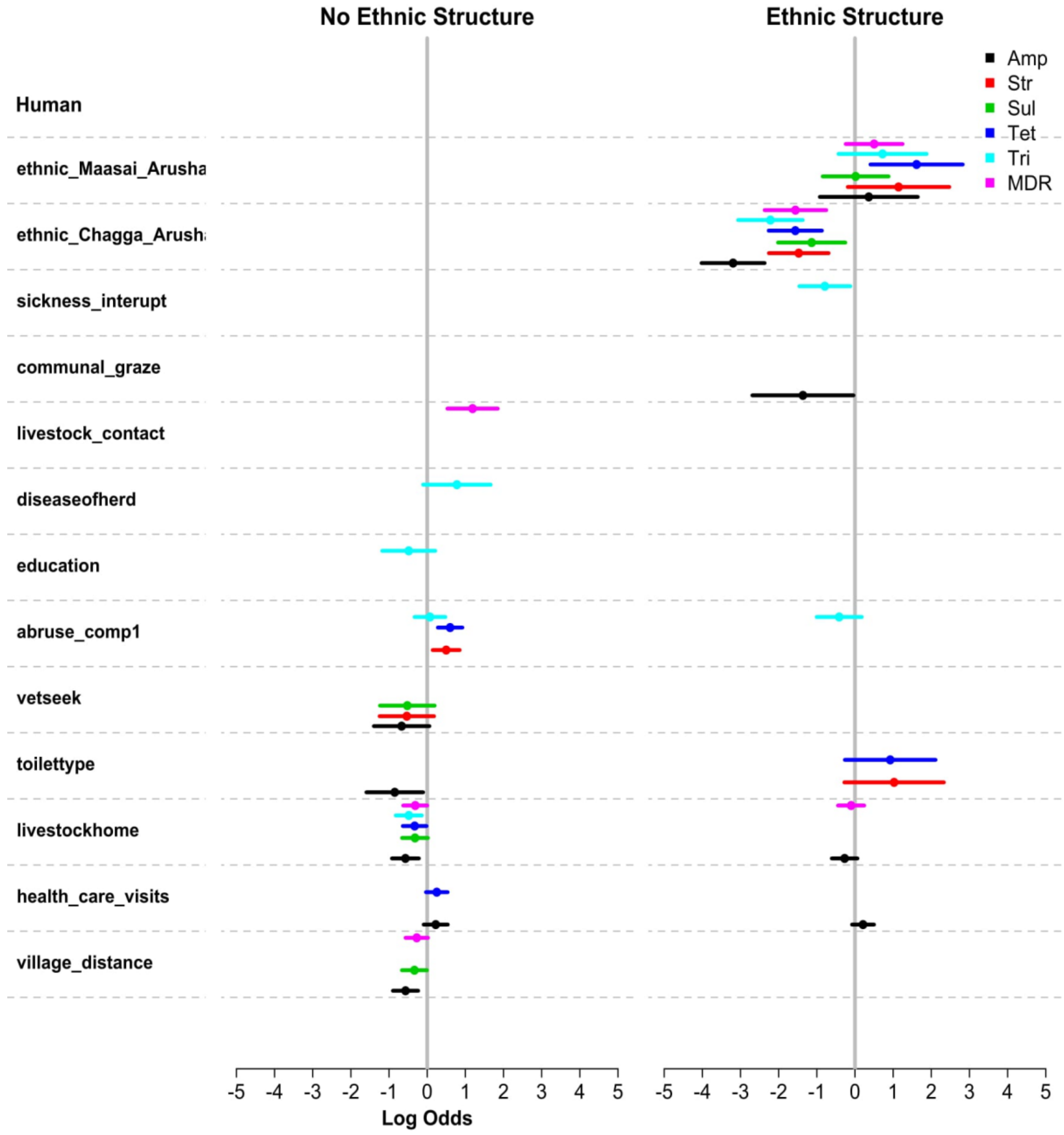

Figure S11. Risk factors for antimicrobial resistant for *E. coli* isolated from people across all ethnic groups (Arusha, Chagga, Maasai). This figure compares models controlling for ethnic group to a model that does not control for ethnic group. Estimated log odds shown as points and 95% confidence interval shown in bars. Colors indicate (Amp)icillin, (Str)eptomycin, (Sul)famethoxazole, (Tet)tracyline, (Tri)methoprime, or MDR (multidrug resistant phenotype).

Table S12. Comparison of average household prevalence of antimicrobial-resistant *E. coli* from fecal samples from Maasai, Chagga and Arusha households. Tabulated *P*-values are from MANOVA analyses.

Ethnic groups	Amp	Cfz	Chl	Cip	Kan	Str	Sul	Tet	Tri
Maasai vs Chagga	<0.0001	0.0005	0.004	0.0006	0.02	<0.0001	<0.0001	<0.0001	<0.0001
Arusha vs Chagga	<0.0001	<0.0001	<0.0001	0.06	0.01	0.0002	<0.0001	<0.0001	<0.0001
Maasai vs Arusha	0.12	0.008	0.001	0.13	1	0.3	0.3	0.003	0.6

Table S13. Comparison of average household prevalence of antibiotic-resistant lactose-fermenting bacteria isolates (N=8,106) from milk samples from Maasai and Arusha households. Tabulated *P*-values are from MANOVA analyses

Ethnic groups	Amp	Cfz	Chl	Cip	Kan	Str	Sul	Tet	Tri
Maasai vs Arusha	0.0582	0.0471	0.0371	0.0001	0.0205	0.0352	0.0028	0.0352	0.0028

References

1 Caudell MA, Quinlan MB, Subbiah M, *et al.* Antimicrobial Use and Veterinary Care among Agro-Pastoralists in Northern Tanzania. *PloS One* 2017; **12**: e0170328.

2 Bej AK, McCarty SC, Atlas R. Detection of coliform bacteria and Escherichia coli by multiplex polymerase chain reaction: comparison with defined substrate and plating methods for water quality monitoring. *Appl Environ Microbiol* 1991; **57**: 2429–32.

3 Park H, Imoto S, Miyano S. Recursive Random Lasso (RRLasso) for Identifying Anti-Cancer Drug Targets. *PloS One* 2015; **10**: e0141869.

4 Meinshausen N, Bühlmann P. Stability selection. *J R Stat Soc Ser B Stat Methodol* 2010; **72**: 417–73.

5 Wang S, Nan B, Rosset S, Zhu J. Random lasso. *Ann Appl Stat* 2011; **5**: 468.

6 Huang S, Marchetti-Bowick M. Summary and discussion of `Stability Selection. .

7 Sabourin JA, Valdar W, Nobel AB. A permutation approach for selecting the penalty parameter in penalized model selection. *Biometrics* 2015; **71**: 1185–94.

8 Groll A, Tutz G. Variable selection for generalized linear mixed models by L1-penalized estimation. *Stat Comput* 2014; **24**: 137–54.

9 Colquhoun D. An investigation of the false discovery rate and the misinterpretation of p-values. *R Soc Open Sci* 2014; **1**. DOI:10.1098/rsos.140216.

10 Lê S, Josse J, Husson F. FactoMineR: an R package for multivariate analysis. *J Stat Softw* 2008; **25**: 1–18.

11 Eberhart LJ, Deringer JR, Brayton KA, Sawant AA, Besser TE, Call DR. Characterization of a novel microcin that kills enterohemorrhagic Escherichia coli O157: H7 and O26. *Appl Environ Microbiol* 2012; **78**: 6592–9.

1. Village *

- Mt. Meru
- Nodonjukin
- Loroi
- Meliot

2. Enumerator *

- Godfrey
- Isaya
- Lemuta
- William

Participant ID Number *

3. Survey Year *

- 2016
- 2015
- 2014
- 2013

Survey Month *

- January
- February
- March
- April
- May
- June
- July
- August
- September
- October
- November
- December

Survey Day *

1
2
3
4
5
6
7
8
9
10
11
12
13
14
15
16
17
18
19
20
21
22
23
24
25
26
27
28
29
30
31

Basic Demographics

4. Gender *

- Male
- Female

5. What is your relationship to the head of the household? *

- Head of household
- Spouse of head of household
- Son of head of household
- In-law
- Hired labor
- Daughter of head of household
- Other

6. Ethnic Group *

- Chagga
- Maasai
- Warusha
- Other

7. How old are you? *

Enter a number.

8. What is your religion? *

- Protestant Christian
- Catholic Christian
- Muslim
- Traditional
- None
- Other

9. Do you read? *

- Yes
- No

10. Highest level of education *

- No formal education
- Some primary school
- Completed primary school
- Some high school
- Completed high school
- Some education beyond high school
- Finished college
- Postgraduate or above

Life History/Demographics

11. Are you married? *

- Yes
- Never
- Divorced
- Widow(er)

12. How many times have you been married? *

- Never
- 1
- 2
- 3
- 4
- 5
- 6
- 7
- 8
- 9
- 10+

13. If married, how old were you when you got married the first time? *
Enter a number.

- 10
- 11
- 12
- 13
- 14
- 15
- 16
- 17
- 18
- 19
- 20
- 21
- 22
- 23
- 24
- 25
- 26
- 27
- 28
- 29
- 30
- 31
- 32
- 33
- 34
- 35
- 36
- 37
- 38
- 39
- 40
- 41
- 42
- 43
- 44
- 45
- 46
- 47
- 48
- 49
- 50
- NEVER MARRIED

14. How many wives do you or your husband have currently? *

- 0
- 1
- 2
- 3
- 4
- 5
- 6
- 7
- 8
- 9
- 10+

15. What is your birth order? *

- First
- Middle
- Last

16. How many siblings do you have? *

	0	1	2	3	4	5	6	7	8	9	10+
Brothers living											
Brothers deceased											
Sisters living											
Sisters deceased											

Children

17. How old were you when your first child was born? *

- No Children
- 10
- 11
- 12
- 13
- 14
- 15
- 16
- 17
- 18
- 19
- 20
- 21
- 22
- 23
- 24
- 25
- 26
- 27
- 28
- 29
- 30
- 31
- 32
- 33
- 34
- 35
- 36
- 37
- 38
- 39
- 40
- 41
- 42
- 43
- 44
- 45
- 46
- 47
- 48
- 49
- 50

18. How many children do you have? *

	0	1	2	3	4	5	6	7	8	9	10	11	12	13	14	15	16+
Sons living *	○	○	○	○	○	○	○	○	○	○	○	○	○	○	○	○	○
Sons deceased *	○	○	○	○	○	○	○	○	○	○	○	○	○	○	○	○	○
Daughters living *	○	○	○	○	○	○	○	○	○	○	○	○	○	○	○	○	○
Daughters deceased *	○	○	○	○	○	○	○	○	○	○	○	○	○	○	○	○	○

19. Did you have any children that didn't survive to one year old? *

○ Yes

○ No

20. Number of persons in household
Enter numbers for all that apply.
If none apply, enter 0 in at least one box.

	Number	Number in School
Children less than school age	Number	Number in School
Females : Primary school age	Number	Number in School
Females : Secondary school age	Number	Number in School
Females : Between 19-65	Number	Number in School
Females : Over 65	Number	Number in School
Males : Primary school age	Number	Number in School
Males : Secondary school age	Number	Number in School
Males : Between 19-65	Number	Number in School
Males : Over 65	Number	Number in School

Health/Health Services

21. What health services do you use for yourself and family? **CHECK ALL THAT APPLY** *

- ☐ Government clinic
- ☐ Private doctor
- ☐ Government hospital
- ☐ Private hospital
- ☐ Local/indigenous practitioners
- ☐ Dispensary
- ☐ None
- ☐ Other (please specify) ____

22. If you can't treat yourself, where do you go FIRST for medical treatment? *

- Government clinic
- Private doctor
- Government hospital
- Private hospital
- Local/indigenous practitioners
- Dispensary
- None
- Other (please specify)

23. If you go to [repeat what they answered above], and they can't help you, then where do you go next for medical treatment? *

This CANNOT be the SAME SELECTION AS 22!

- Government clinic
- Private doctor
- Govt hospital
- Private hospital
- Local/indigenous practitioners
- None, I only have ONE option
- Other (please specify)

24. What was the most recent illness for a person in the household? *

25. Where did you USUALLY get medicine come from? *

- Government clinic/ hospital
- Private doctor
- Private clinic/ hospital
- Pharmacy
- Friend or neighbor
- N/A
- Other (please specify)

26. What was the most recent exotic medicine someone in your household used? *

- I have not used an exotic medicine recently
- Yes, but I don't know the name
- Yes, I have used the name is?

27. How many times has someone used antibiotics in your household in the last year?

- 0
- 1-3 times
- 4-6 times
- 7-10 times
- Over 10 times

28. Where did you USUALLY get ANTIBIOTICS come from? *

- Government clinic/ hospital
- Private doctor
- Private clinic/ hospital
- Pharmacy
- Friend or neighbor
- N/A
- Other (please specify) ____

29. Are children less than 5 years old in the household currently vaccinated against one or more diseases? *

- Yes
- No
- No children under 5 years

30. How much time does it take to travel to the health center or hospital that you use (ONE WAY)?

Enter numbers. Only need to fill in one box.

minutes by foot ____

minutes by vehicle ____

31. How many visits to a health clinic or hospital were made by household members in the last 3 months (sum of everyone in house)? *

- 0-1 time
- 2-4 times
- 5-10 times
- 11-20 times
- 20+ times

Social - Household Information

32. How many dwellings? *
Enter numbers.

- 1-3
- 3-6
- 7-10
- Over 10

33. Animal House

	Yes	No
Cattle		
Goats and Sheep		
Chicken		
Dog		

34. The house of the respondent is: *

- On own land
- On communal land
- Other ______

35. Toilet location? *

- Inside the house
- Outside the house
- Both
- None

36. What type of toilet? *

- [ ] Flush toilet
- [ ] Pit toilet
- [ ] Trench
- [ ] Bush in dedicated location
- [ ] Bush in general
- [ ] Other ______

37. Electricity –Household inventory *

- None
- Grid (this means a power line is connected to house
- Off grid (but available via local production such as solar panels)

38. How many of these does your household have? *

	0	1	2	3	4	5+
Radio Functioning	○	○	○	○	○	○
Television Functioning	○	○	○	○	○	○
Refrigerator Functioning	○	○	○	○	○	○
Bicycle : Functioning	○	○	○	○	○	○
Motorcycle/Scooter : Functioning	○	○	○	○	○	○
Car/Truck : Functioning	○	○	○	○	○	○
Cell Phone : Functioning	○	○	○	○	○	○
Horse Cart: Functioning	○	○	○	○	○	○
Plough : Functioning	○	○	○	○	○	○
Syringe : Functioning	○	○	○	○	○	○
Needles : Functioning	○	○	○	○	○	○
M'kokoteli(Wheel Barrow)	○	○	○	○	○	○

39. Where do you get your water? **CHECK ALL THAT APPLY** *

- ☐ Communal well
- ☐ Private well
- ☐ River water
- ☐ Lake or water hole
- ☐ Rain water or seasonal stream
- ☐ Household Stand pipe
- ☐ Nieghbor Standpipe
- ☐ Pay for delivery
- ☐ Cistern
- ☐ Other (please specify) ____________

40. Where do you usually get your water? *

- Communal well
- Private well
- River water
- Lake or impoundment
- Rain water seasonal stream
- Household Stand pipe
- Neighbor Standpipe
- Pay for delivery
- Other (please specify)

41. Do livestock or wildlife drink from the same water source? For example, livestock and humans all drink from standpipe *

- No, water only for people
- Livestock drink the same water
- Wildlife drink the same water
- Both livestock and wildlife drink the same water

42. Do you treat drinking water? *

- No treatment
- Boil every time
- Boil some of the time
- Chemical treatment
- Filter
- Other ______

Consumption

43. How often do you eat meat in your meals? *

	Every meal	Once per day	More than once a week	Less than once per week	Less than once per month	Special occasions	Never
Beef *							
Sheep *							
Goat *							
Pig meat *							
Poultry *							

44. Quantity of cow's milk consumed by household per day

	Quantity
Home produced milk	Quantity
Purchased milk	Quantity
Home produced butter	Quantity
Purchased butter	Quantity
Home produced eggs	Quantity
Purchased eggs	Quantity

Economic - Crop Enterprise, Land

45. **FOR THE LAST HARVEST** what types of crops did you grow?
If NO CROPS, enter NA for at least one entry.

	Crop name	Area planted	Draught power used (Yes/No)	Draught power used (Type)	Fertilizer used (Yes/No)	Fertilizer used (Type)	Crop output use (Home Consumption)
Crop 1	Crop name	Area planted	Draught power used (Yes/No)	Draught power used (Type)	Fertilizer used (Yes/No)	Fertilizer used (Type)	Crop output use (Home Consumption)
Crop 2	Crop name	Area planted	Draught power used (Yes/No)	Draught power used (Type)	Fertilizer used (Yes/No)	Fertilizer used (Type)	Crop output use (Home Consumption)
Crop 3	Crop name	Area planted	Draught power used (Yes/No)	Draught power used (Type)	Fertilizer used (Yes/No)	Fertilizer used (Type)	Crop output use (Home Consumption)
Crop 4	Crop name	Area planted	Draught power used (Yes/No)	Draught power used (Type)	Fertilizer used (Yes/No)	Fertilizer used (Type)	Crop output use (Home Consumption)
Crop 5	Crop name	Area planted	Draught power used (Yes/No)	Draught power used (Type)	Fertilizer used (Yes/No)	Fertilizer used (Type)	Crop output use (Home Consumption)

46. Total crop production expenditures for the most recent complete growing season
If NO CROPS, enter 0 for at least one entry.

	Cost (in TZS)
Seed	Cost (in TZS)
Fertilizer	Cost (in TZS)
Herbicide	Cost (in TZS)
Pesticide	Cost (in TZS)
Tractor Rental	Cost (in TZS)
Other 1 (specify below)	Cost (in TZS)
Other 2 (specify below)	Cost (in TZS)
Other 3 (specify below)	Cost (in TZS)

47. Did you lose 1/4 or more of your crops in the following years? *

2014	Yes, I lost 1/4 of my crops<br>Yes, I lost 1/2 of my crops<br>Yes, I lost 3/4 of my crops<br>Yes, I lost ALL of my crops<br>No, I did not lose any crops<br>No, I don't GROW ANY CROPS<br>I don't remember
2013	Yes, I lost 1/4 of my crops<br>Yes, I lost 1/2 of my crops<br>Yes, I lost 3/4 of my crops<br>Yes, I lost ALL of my crops<br>No, I did not lose any crops<br>No, I don't GROW ANY CROPS<br>I don't remember
2012	Yes, I lost 1/4 of my crops<br>Yes, I lost 1/2 of my crops<br>Yes, I lost 3/4 of my crops<br>Yes, I lost ALL of my crops<br>No, I did not lose any crops<br>No, I don't GROW ANY CROPS<br>I don't remember
2011	Yes, I lost 1/4 of my crops<br>Yes, I lost 1/2 of my crops<br>Yes, I lost 3/4 of my crops<br>Yes, I lost ALL of my crops<br>No, I did not lose any crops<br>No, I don't GROW ANY CROPS<br>I don't remember
2010	Yes, I lost 1/4 of my crops<br>Yes, I lost 1/2 of my crops<br>Yes, I lost 3/4 of my crops<br>Yes, I lost ALL of my crops<br>No, I did not lose any crops<br>No, I don't GROW ANY CROPS<br>I don't remember

48. If you lost a 1/4 or more of your crops in the following years, what was the major reason? *

2014
- Drought
- Pests or Disease
- Bad Seeds or Not Enough Fertilizer
- Animals Ate
- Didn't take care of the field
- I don't have crops
- I didn't lose any crops that year
- I don't remember

2013
- Drought
- Pests or Disease
- Bad Seeds or Not Enough Fertilizer
- Animals Ate
- Didn't take care of the field
- I don't have crops
- I didn't lose any crops that year
- I don't remember

2012
- Drought
- Pests or Disease
- Bad Seeds or Not Enough Fertilizer
- Animals Ate
- Didn't take care of the field
- I don't have crops
- I didn't lose any crops that year
- I don't remember

2011
- Drought
- Pests or Disease
- Bad Seeds or Not Enough Fertilizer
- Animals Ate
- Didn't take care of the field
- I don't have crops
- I didn't lose any crops that year
- I don't remember

2010
- Drought
- Pests or Disease
- Bad Seeds or Not Enough Fertilizer
- Animals Ate
- Didn't take care of the field

I don't have crops
I didn't lose any crops that year
I don't remember

Livestock Enterprise, Assets

49. Cattle
Enter numbers.
If NO CATTLE, enter 0 for at least one entry.

	Adult (Number)	Young (Number)
Kept in a boma in homestead part of all of the day	Adult (Number)	Young (Number)
Moves in and out of the homestead	Adult (Number)	Young (Number)
Herded away from the homestead	Adult (Number)	Young (Number)

50. Sheep & goats

Enter numbers.

If NO GOATS AND SHEEP, enter 0 for at least one entry.

	Adult (Number)	Young (Number)
Kept in a boma in homestead part of all of the day	Adult (Number)	Young (Number)
Moves in and out of the homestead	Adult (Number)	Young (Number)
Herded away from the homestead	Adult (Number)	Young (Number)

51. Donkeys

Enter numbers.

If NO DONKEYS, enter 0 for at least one entry.

	Adult (Number)	Young (Number)
Kept in a boma in homestead part of all of the day	Adult (Number)	Young (Number)
Moves in and out of the homestead	Adult (Number)	Young (Number)
Herded away from the homestead	Adult (Number)	Young (Number)

52. Pigs
Enter numbers.
If NO PIGS, enter 0 for at least one entry.

	Animals (Number)	Purchase in last year? If Yes, put number	Source of Purchase
adult	Animals (Number)	Purchase in last year? If Yes, put number	Source of Purchase
young	Animals (Number)	Purchase in last year? If Yes, put number	Source of Purchase

53. Poultry system

	Birds (Number)	Housing?	Purchase in last year, if yes put #
Free range	Birds (Number)	Housing?	Purchase in last year, if yes put #

54. number of eggs produced per flock/day? *
Enter a number.

eggs/flock/day

55. Average amount of eggs sold by the household *
Enter a number.

eggs per day

56. How many animals have you purchased in the last year? *

Cattle

Sheep/goats

Donkeys

57. How many animals have you sold in the last year? *

Cattle

Sheep/goats

Donkeys

58. Where do you sell cattle and small livestock? **(Check ALL that apply)** *

- [ ] Local market
- [ ] Regional market
- [ ] Neighbors
- [ ] Butcher/abattoir
- [ ] I don't sell cattle
- [ ] I don't own any cattle
- [ ] Other (specify)

59. When you sell livestock how far do you usually travel? *

- 1 hour or less
- More than 1 hour less than 1 day
- More than 1 day
- More than 2 days
- N/A

60. When you purchase livestock how far do you usually travel? *

- 1 hour or less
- More than 1 hour less than 1 day
- More than 1 day
- More than 2 days
- N/A

61. Does someone outside your household manage your livestock for you? *

- Yes
- No

62. If someone else manages your livestock outside of your household, why? *

- Too many to manage alone
- Other person needed livestock for milk
- N/A
- Other (specify) ______

63. How many animals do you currently manage for someone else? *
Enter numbers for all that apply.
If none apply, enter 0 for at least one entry.

Cattle ______

Goats ______

Sheep ______

Donkeys ______

Horses ______

Other ______

64. Why are you keeping stock for someone else? (SKIP IF NOT KEEPING STOCK FOR SOMEONE ELSE) *

- Owner had too many to manage alone
- Needed livestock for milk
- Kumharia
- N/A
- Other (specify) ______

65. How much land does the household graze and not share with other livestock holders for grazing? *

Enter the number and the units.

- ○ Yes, write in number and units ________ *
- ○ None

66. Does your family have access to common grazing land? *

- ○ Yes
- ○ No

67. What factors keep you from increasing your herd size? (CHECK ALL THAT APPLY) *

- ☐ Lack of money
- ☐ Lack of land
- ☐ Lack of water
- ☐ Lack of labor
- ☐ Don't want any more
- ☐ Too expensive to maintain
- ☐ Disease limitations
- ☐ N/A
- ☐ Other (specify) ________

68. How often do your livestock come into contact with livestock from other villages/communities when grazing? *

- Never
- Every day
- Once or more per week
- Once or more per month
- Less than once per month

Cattle Management

69. How many times in a normal year do you move your livestock? *

- 0
- 1
- 2
- 3
- 4
- 5
- 6+
- NA, I have no livestock

70. How many times do you move your livestock in a dry year? *

- 0
- 1
- 2
- 3
- 4
- 5
- 6+
- NA, I have no livestock

71. How far do you move your livestock in a normal year? *

- Less than one day walk
- One to two days
- Three to four days
- More than four days
- N/A

72. How far do you move your livestock in a dry year? *

- Less than one day walk
- One to two days
- Three to four days
- More than four days
- N/A

73. Is this year a dry year, normal year, or wet year? *

- Dry year
- Normal year
- Wet year

Economic - Livestock Health and Loss

74. Did you lose one-quarter or more of your livestock in the following years? *

For example, if their herd was 4 and they lost 1 cow or sheep then say "yes"

2014

- Yes, I lost 1/4 of my herd
- Yes, I lost 1/2 of my herd
- Yes, I lost 3/4 of my herd
- Yes, I lost ALL of my herd
- No, I didn't lose more than 1/4
- No, I don't have any livestock
- I don't remember

2013

- Yes, I lost 1/4 of my herd
- Yes, I lost 1/2 of my herd
- Yes, I lost 3/4 of my herd
- Yes, I lost ALL of my herd
- No, I didn't lose more than 1/4
- No, I don't have any livestock
- I don't remember

2012

- Yes, I lost 1/4 of my herd
- Yes, I lost 1/2 of my herd
- Yes, I lost 3/4 of my herd
- Yes, I lost ALL of my herd
- No, I didn't lose more than 1/4
- No, I don't have any livestock
- I don't remember

2011

- Yes, I lost 1/4 of my herd
- Yes, I lost 1/2 of my herd
- Yes, I lost 3/4 of my herd
- Yes, I lost ALL of my herd
- No, I didn't lose more than 1/4
- No, I don't have any livestock
- I don't remember

2010

- Yes, I lost 1/4 of my herd
- Yes, I lost 1/2 of my herd
- Yes, I lost 3/4 of my herd
- Yes, I lost ALL of my herd
- No, I didn't lose more than 1/4
- No, I don't have any livestock
- I don't remember

75. If you lost 1/4 or more of your herds in the following years, what was the major reason? *

2014	Drought<br>Pests or Disease<br>I did not lose 1/4 of my herd that year<br>I don't have any livestock<br>I don't remember
2013	Drought<br>Pests or Disease<br>I did not lose 1/4 of my herd that year<br>I don't have any livestock<br>I don't remember
2012	Drought<br>Pests or Disease<br>I did not lose 1/4 of my herd that year<br>I don't have any livestock<br>I don't remember
2011	Drought<br>Pests or Disease<br>I did not lose 1/4 of my herd that year<br>I don't have any livestock<br>I don't remember
2010	Drought<br>Pests or Disease<br>I did not lose 1/4 of my herd that year<br>I don't have any livestock<br>I don't remember

76. What animal health services do you use for livestock? **CHECK ALL THAT APPLY** *

- ☐ Ag extension officer
- ☐ Government vet
- ☐ Private vet
- ☐ Drug shop
- ☐ Indigenous healer
- ☐ None
- ☐ Other (specify) ____________

77. Describe the most recent illness in your herd (SKIP IF NO RECENT ILLNESS)

	Animal	Illness	Symptoms	Treatment	Duration	Number sick	Recovered
Most recent	Animal	Illness	Symptoms	Treatment	Duration	Number sick	Recovered
Next most recent	Animal	Illness	Symptoms	Treatment	Duration	Number sick	Recovered

78. Are your animals vaccinated against any diseases? (SKIP IF NO ANIMALS ARE VACCINATED)

List for which diseases animals are vaccinated.

	Disease 1	Disease 2	Disease 3	Disease 4	Other diseases
Cattle	Disease 1	Disease 2	Disease 3	Disease 4	Other diseases
Smallstock	Disease 1	Disease 2	Disease 3	Disease 4	Other diseases
Dogs	Disease 1	Disease 2	Disease 3	Disease 4	Other diseases

79. Where do you get vaccines for livestock? *

- Veterinarian
- Animal drug shop
- Agricultural extension
- Friends
- N/A, the HOUSEHOLD does not purchase vaccines
- Don't Know
- Other (specify)

80. Number of calves born dead in the last year from sick cows? *

Enter a number.

81. Number of kids born dead in last year from sick mother? *

Enter a number.

82. How do you dispose of a dead animal? *

- Eat
- Skin and bury
- Bury without skinning
- Leave it
- Burn it
- Cook for dogs and pigs
- N/A

83. When you treat an animal do you use the milk or meat from it? *

- Immediately
- After 3 days
- After 10 days
- When the animal is healthy
- N/A

84. Has sickness in your herd reduced the time available for other business/work in the last year? *

- Yes
- No

85. Has any family member become sick or died because of eating product from sick animal? *

- Yes
- No

Exotic Medicines for Livestock

86. Do you keep exotic medicines FOR LIVESTOCK at home? *

- Yes
- No

87. What exotic medicines **FOR LIVESTOCK** do you keep on hand at home?

	Medicine	Brand	Where it was purchased	When it was purchased	Use by date (EXPIRATION)
Medicine 1	Medicine	Brand	Where it was purchased	When it was purchased	Use by date (EXPIRATION)
Medicine 2	Medicine	Brand	Where it was purchased	When it was purchased	Use by date (EXPIRATION)
Medicine 3	Medicine	Brand	Where it was purchased	When it was purchased	Use by date (EXPIRATION)
Medicine 4	Medicine	Brand	Where it was purchased	When it was purchased	Use by date (EXPIRATION)
Medicine 5	Medicine	Brand	Where it was purchased	When it was purchased	Use by date (EXPIRATION)
Medicine 6	Medicine	Brand	Where it was purchased	When it was purchased	Use by date (EXPIRATION)
Medicine 7	Medicine	Brand	Where it was purchased	When it was purchased	Use by date (EXPIRATION)
Medicine 8	Medicine	Brand	Where it was purchased	When it was purchased	Use by date (EXPIRATION)

88. Where do you get exotic medicines for livestock? *

- Veterinarian
- Animal drug shop
- Agricultural extension
- Friends
- N/A, the HOUSEHOLD does not purchase exotic medicines
- Other (specify) ____

Cattle Sickness

89. What do you do to avoid disease/outbreak in livestock? **CHECK ALL THAT APPLY** *
Select all that apply.

- [ ] Keep calves separate
- [ ] Make a shed or pen to prevent contact from other domestic and wild animals
- [ ] Keep new cattle separately
- [ ] Graze sick cattle separately
- [ ] Feed supplementation
- [ ] Buy new cattle from local area
- [ ] Vaccination
- [ ] Treatment (Animal Health)
- [ ] Treatment (traditional)
- [ ] Spraying
- [ ] Do nothing
- [ ] Other (specify) ____

90. Do you change where you graze when your livestock to avoid sickness? *

- Yes
- No
- N/A, I don't have any cows
- N/A, my cows are zero grazing

91. Do you ever give your livestock medicine? *

- Yes, I give them exotic and traditional medicine
- Yes, but I only give them traditional medicine NOT exotic medicine
- Yes, but I only give them exotic medicine NOT traditional medicine
- No, I do not give them any medicine.

92. How much do you use per dose for a full grown cow?

	Length of treatment (days)	dose for adult cow	Don't Know, Vet gives drugs
Pen-strep	Length of treatment (days)	dose for adult cow	Don't Know, Vet gives drugs
Basulfa	Length of treatment (days)	dose for adult cow	Don't Know, Vet gives drugs
Bamisola	Length of treatment (days)	dose for adult cow	Don't Know, Vet gives drugs
Dininabazen	Length of treatment (days)	dose for adult cow	Don't Know, Vet gives drugs
Parvexon	Length of treatment (days)	dose for adult cow	Don't Know, Vet gives drugs
Alfamec	Length of treatment (days)	dose for adult cow	Don't Know, Vet gives drugs
Ivermectine	Length of treatment (days)	dose for adult cow	Don't Know, Vet gives drugs
Inomazene	Length of treatment (days)	dose for adult cow	Don't Know, Vet gives drugs

(untitled)

93. Does anyone in the household milk cows *

- Yes
- No

94. How much milk do you get from **ONE COW** in the

Dry Season
- Under 1 liter
- 1 liter
- 1-2 liter
- 2 liters
- more than 2 liters

Wet Season
- Under 1 liter
- 1 liter
- 1-2 liter
- 2 liters
- more than 2 liters

95. How many cows does THE HOUSEHOLD milk in the

HOUSEHOLD INCLUDES ALL WIVES

Dry Season

- 1-2
- 2-4
- 5-10
- 11-20
- 21-40
- 41-60
- Over 60

Wet Season

- 1-2
- 2-4
- 5-10
- 11-20
- 21-40
- 41-60
- Over 60

96. Average amount of cow milk sold by the household *
Enter a number.

[ ] number of liters/day

97. Average amount of butter sold by the household *
Enter a number.

[ ] liters/ market day

98. Does the housheold milk goats/sheep *

- Yes
- No

99. How much milk do you get from **ONE GOAT/SHEEP** in the:

Dry Season

- Under 1 liter
- 1 liter
- 1-2 liter
- 2 liters
- more than 2 liters

Wet Season

- Under 1 liter
- 1 liter
- 1-2 liter
- 2 liters
- more than 2 liters

100. How many goats/sheep does THE HOUSEHOLD milk in the

HOUSEHOLD INCLUDES ALL WIVES

Dry Season

- 1-2
- 2-4
- 5-10
- 11-20
- 21-40
- 41-60
- Over 60

Wet Season

- 1-2
- 2-4
- 5-10
- 11-20
- 21-40
- 41-60
- Over 60

101. Average amount of goat milk sold by the household *
Enter a number.

[ ] liters/ day

102. Where do you normally sell milk or butter?

- [ ] Local market
- [ ] Regional market
- [ ] Neighbors
- [ ] Shop keeper
- [ ] N/A
- [ ] Other (specify) [ ]

(untitled)

103. Select YES if anyone in the household milk cows OR goats/sheep

- Yes
- No

104. How many times a day do you milk cows or goats?

- 1
- 2
- 3
- 4 or more

105. Do you clean the enyewa of the cows/sheep/goats before milking?

- Yes
- No

106. Do you DRINK/COOK with MILK from? *

SICK COWS	Yes<br>No<br>N/A, I don't have any milk producing livestock
SICK GOATS/SHEEP	Yes<br>No<br>N/A, I don't have any milk producing livestock

107. Do you SELL MILK from? *

SICK COWS
- Yes
- No
- N/A, I don't have any milk producing livestock

SICK GOATS/SHEEP
- Yes
- No
- N/A, I don't have any milk producing livestock

108. Do you stop selling or consuming milk during treatment with exotic medicines *

- Yes
- No
- N/A

109. Is there a decrease in milk production from sick cows compared to healthy cows? *

- Yes
- No
- No milking of sick cows
- N/A , I don't have any cows

110. Referring to the previous question, If yes, by how much? (SKIP IF NO DECREASE IN MILK PRODUCTION) *

Enter a number.

[ ] liters/day

111. Do you have seperate COLLECTION CONTAINERS for DIFFERENT COWS/SHEEP?GOATS?

- YES, each animal has a different container
- NO, I use the same container for many animals
- No, I onle have ONE milking animal

112. If one of your milking animals is sick, do you stop milking and clean the collection container BEFORE milking any other animals?

- Yes, every time
- Yes, but only some of the times
- No

113. How often do you clean your calabash/container used for COLLECTING cow and sheep milk

- Only in the morning
- Only in the evening
- In the morning and in the evening
- A few times per week
- I never clean my collection containers

114. What do you clean your milk containers with? **CHECK ALL THAT APPLY**

	Water	Cow Urine
Collection Containers	☐	☐
Storage Containers	☐	☐

115. What CONTAINER do you STORE milk in? **CHECK ALL THAT APPLY**

- [ ] Calabash
- [ ] Plastic Container
- [ ] Metal Pots
- [ ] Other (required) ______ *

116. How many hours do you USUALLY store milk after morning or evening milking before it is all gone?

- ○ Under 1 hr
- ○ 1-2
- ○ 3-4
- ○ 5-7
- ○ 8-12
- ○ 12-24
- ○ More than one day

117. How many times do you bring the milk to boil?

- ○ 1
- ○ 2
- ○ 3
- ○ 4
- ○ Over 4

118. Do you boil milk before putting in calabash to make sour milk?

- Yes, always
- Yes, but only some of the itme
- No, I do not boil the milk
- I do not make sour milk

119. How many days do you keep sour milk before cleaning the calabash?

- Under 1 day
- About 1 day
- 1-2 days
- 3-4 days
- 5-6 days
- A week or more
- I do not make sour milk

120. If you do not boil all of the milk, why do you not boil all of the milk? **CHECK ALL THAT APPLY**

- I do not boil the milk I sell
- Boiled milk tastes bad
- Boiled milk hurts the vitamines in milk
- I do not boil milk
- Other (required) ____________ *

121. Do you use the SAME CONTAINERS to collect blood milk and urine

- Yes
- No, I use different containers
- No, I do not collect blood or urine from animals

DAAAGS

122. Do you own any dogs?

- Yes, put how many ______ *
- No

123. How often do you feed your dogs?

- almost everyday
- a few times per week
- a few times per week
- I never feed them, they find food for themselves

124. If you feed your dogs, what do you feed them. Ask SEPERATELY FOR DRY AND WET SEASONS. CHECK ALL THAT APPLY

	Porridge with Milk	Porridge with Water	Milk Water Mixed	Only Milk	Only Water	Left over human food
Dry Season	☐	☐	☐	☐	☐	☐
Wet Season	☐	☐	☐	☐	☐	☐

125. How long do you cook the porridge for dogs?

- Under 5 minutes
- 5-10 minutes
- over 10 minutes

126. Do you cook your porridge for dogs FOR LESS time than you cook porridge for humans?

- Yes
- No

127. If you cook porridge for dogs FOR LESS time, why?

128. If you don't drink milk from sick cows, do you still give it to dogs?

- Yes
- No

129. Have you ever seen dogs EAT the poop of. CHECK ALL THAT APPLY

- Humans
- Other Dogs
- Cattle
- Sheep/Goat

Ego Network

130. In the last year, how many DIFFERENT people did you RECEIVE livestock from. This includes buying, loans, marriage payments, and gifts *

131. In the last year, about how many DIFFERENT people did you GIVE livestock to. This includes selling, loans, marriage payments, and gifts *

132. Please think about the last three times you BOUGHT OR SOLD cattle, who did you BUY or SELL them too *

Exchange 1
- Kin
- Friend
- Livestock Trader
- NA

Exchange 2
- Kin
- Friend
- Livestock Trader
- NA

Exchange 3
- Kin
- Friend
- Livestock Trader
- NA

133. List up to 3 water sources your livestock use in a year.

	Name of Source
Water A	Name of Source
Water B	Name of Source
Water C	Name of Source

134. How often do your livestock use the sources

Water A
- almost everyday
- almost once per week
- about once per month

Water B
- almost everyday
- almost once per week
- about once per month

Water C
- almost everyday
- almost once per week
- about once per month

135. Do your livestock come in contact with other livestock at these sources

Water A

- Yes, many other animals
- Yes, but only a few households
- No, this source is in the household
- No, I take water from source and bring to cattle

Water B

- Yes, many other animals
- Yes, but only a few households
- No, this source is in the household
- No, I take water from source and bring to cattle

Water C

- Yes, many other animals
- Yes, but only a few households
- No, this source is in the household
- No, I take water from source and bring to cattle

136. List up to 3 markets you have bought livestock at

	Name of Source
Market A	Name of Source
Market B	Name of Source
Market C	Name of Source

137. For the year, how many cattle did you BUY at the market

Market A
- 0
- 1
- 2-4
- 5-10
- 10-30
- 31-50
- Over 50

Market B
- 0
- 1
- 2-4
- 5-10
- 10-30
- 31-50
- Over 50

Market C
- 0
- 1
- 2-4
- 5-10
- 10-30
- 31-50
- Over 50

138. For the year, how many cattle did you SELL at the market

Market A
- 0
- 1
- 2-4
- 5-10
- 10-30
- 31-50
- Over 50

Market B
- 0
- 1
- 2-4
- 5-10
- 10-30
- 31-50
- Over 50

Market C
- 0
- 1
- 2-4
- 5-10
- 10-30
- 31-50
- Over 50

Information on wage labor

139. In the last three years, has anyone in the household been employed in wage labor? This means the person is paid at an hourly rate *

- Yes
- NO

140. Please think of the last three people IN THE HOUSEHOLD who worked wage labor. Who?

Person 1

- No person has worked wage labor
- Household Head
- Spouse of Household Head
- Child of household head
- Sibling of Household Head
- Parents of Household Head
- Cousin
- Friend

Person 2

- No person has worked wage labor
- Household Head
- Spouse of Household Head
- Child of household head
- Sibling of Household Head
- Parents of Household Head
- Cousin
- Friend

Person 3

- No person has worked wage labor
- Household Head
- Spouse of Household Head
- Child of household head
- Sibling of Household Head
- Parents of Household Head
- Cousin
- Friend

141. Where did the people go for wage labor?

Person 1
- close to the village
- In an urban area
- many kilometers away

Person 2
- close to the village
- In an urban area
- many kilometers away

Person 3
- close to the village
- In an urban area
- many kilometers away

142. How long did the people work in wage labor?

Person 1
- A few weeks
- A few months
- Many months

Person 2
- A few weeks
- A few months
- Many months

Person 3
- A few weeks
- A few months
- Many months

143. What type of wage labor did the person do. For example, security, work for tourist company, get paid to weed field

Person 1

Person 2

Person 3

144. In the last three years, has anyone in the household made money from self-employment, such as boda-boda, selling charcoal, or selling matunda from garden? *

- Yes
- NO

145. Please think of the last three people IN THE HOUSEHOLD who worked self-employed. Who were they?

Person 1
- Household Head
- Spouse of Household Head
- Child of household head
- Sibling of Household Head
- Parents of Household Head
- Cousin
- Friend

Person 2
- Household Head
- Spouse of Household Head
- Child of household head
- Sibling of Household Head
- Parents of Household Head
- Cousin
- Friend

Person 3
- Household Head
- Spouse of Household Head
- Child of household head
- Sibling of Household Head
- Parents of Household Head
- Cousin
- Friend

146. What type of self-employment did the person do? For example, drive a boda-boda, sell charcoal, or or vegetables/eggs from garden

Person 1

Person 2

Person 3

147. In the last three years, has anyone IN THE HOUSEHOLD made money from salary labor? Salay Labor means paid per month, for example, working for the government *

- Yes
- NO

148. Please think of the last three people who worked salary labor IN THE HOUSEHOLD, who were they?

Person 1

- No person has worked in salary labor
- Household Head
- Spouse of Household Head
- Child of household head
- Sibling of Household Head
- Parents of Household Head
- Cousin
- Friend

Person 2

- No person has worked in salary labor
- Household Head
- Spouse of Household Head
- Child of household head
- Sibling of Household Head
- Parents of Household Head
- Cousin
- Friend

Person 3

- No person has worked in salary labor
- Household Head
- Spouse of Household Head
- Child of household head
- Sibling of Household Head
- Parents of Household Head
- Cousin
- Friend

149. How often do the people work in salary labor?

Person 1
- A few weeks
- A few months
- Many months

Person 2
- A few weeks
- A few months
- Many months

Person 3
- A few weeks
- A few months
- Many months

150. What type of salary labor did the person do. For example, work for government or NGO

Person 1 ____________________

Person 2 ____________________

Person 3 ____________________

151. Has any person working in SALARY or WAGE LABOR AWAY FROM THE VILLAGE, sent money back to the household? *

Person 1

- No person has sent back money
- Household Head
- Spouse of Household Head
- Child of household head
- Sibling of Household Head
- Parents of Household Head
- Cousin
- Friend

Person 2

- No person has sent back money
- Household Head
- Spouse of Household Head
- Child of household head
- Sibling of Household Head
- Parents of Household Head
- Cousin
- Friend

Person 3

- No person has sent back money
- Household Head
- Spouse of Household Head
- Child of household head
- Sibling of Household Head
- Parents of Household Head
- Cousin
- Friend

152. If someone working in wage or salary labor away from household sent money home, how often did they send home?

Person 1	almost every week<br>almost every month<br>only a few times a year
Person 2	almost every week<br>almost every month<br>only a few times a year
Person 3	almost every week<br>almost every month<br>only a few times a year

153. What is the total household wage income per month on average from labor, INCLUDING WAGE, SALARY. SMALL BUSINESS and NOT from livestock or crops? *

Enter a number.

- 0
- 1- 25,000
- 25,000-100,000
- 100,000-200,000
- 200,000-400,000
- 400,000-700,000
- 700,000-1,000,000
- Over 1 million
- Does not want to answer
- Does not know

154. Does any household member maintain a savings account? *

- Bank
- Mpesa
- None

155. What is the current household savings balance? (SKIP IF NO BANKING ACCOUNT) *
Enter a number.

- 0
- 1- 25,000
- 25,000-100,000
- 100,000-200,000
- 200,000-400,000
- 400,000-700,000
- 700,000-1,000,000
- Over 1 million
- Does not want to answer
- Does not know

156. In the LAST YEAR did you receive any support for feeding the household from any NGO/Government? *

- Yes, write in name of source (agency, NGO) ____________
- No

157. Has any household member taken **a loan or loans** in the last 2 years? *

- Yes
- No
- Don't know

158. Why were the loan (s) taken? **CHECK ALL THAT APPLY** *

- Buy food
- Housing
- Transportation
- School fees
- Health care
- Livestock purchase
- Business
- N/A
- Other

Diversification

159. In the last 3 years, have you changed **the size** of your herd or **species composition** because of changes in the environment, such as climate changes. *

- Yes
- No
- No, I did not own any livestock in the past three years

160. In the last 3 years, have you increased the amount of livestock you sold? *

- Yes
- No
- No, I did not own any livestock in last three years

161. In the last 3 years, have you changed the types of crops you grow because of climate changes or to make more money? *

- Yes, because of climate change
- Yes, to make more money
- Yes, because of climate change AND money
- No
- No, I did not have any crops in last 3 years

162. In the last 3 years, have you increased inputs (fertilizer, labor) to increase agricultural output (yield)? *

- Yes
- No
- No, I did not have any crops in last 3 years

Diversification

163. In the Last Year What percent of household income was from Livestock and Crops OR Labor/Assistance *

- 100% Livestock and Crops
- 90% Livestock and Crops and 10% Labor
- 75% Livestock and Crops and 25% Labor
- 50% Livestock and Crops and 50% Labor
- 25% Livestock and Crops and 75% Labor
- 10% Livestock and Crops and 90% Labor
- 100% Labor and assistance

164. From the money earned by livestock and crops, what percent is from livestock and from crops *

- 100% Livestock
- 90% Livestock and 10% Crops
- 75% Livestock and 25% Crops
- 50% Livestock and 50% Crops
- 25% Livestock and 75% Crops
- 10% Livestock and 90% Crops
- 100% Crops
- NA, I get ALL income from Labor/Assistance

VERIFY

165. Did you get a GPS point and label the point the household id number *

- Yes
- NO

166. Did you get a milk sample (fresh, sour, cattle and goat) if they have this milk? If they did have milk, make sure you put ID Number, Date, Time the Milking (morning or evening), Time you collected, and Type of Container used (calabash, plastic container) *

- Yes
- No

167. Did you get poop samples (human, cattle, sheep, dog, chicken, donkey) and make sure to put ID, DATE, Time of COllection, Species and OPEN/ENCLOSURE? *

- Yes
- No

Original Letter of Transmittal

Click here to download Necessary Additional Data: Letter of transmittal EEID.pdf
